# Epidemiological investigation of coccidiosis and associated risk factors in broiler chickens immunized with live anticoccidial vaccines in China

**DOI:** 10.3389/fvets.2024.1375026

**Published:** 2024-03-19

**Authors:** Shenquan Liao, Xuhui Lin, Qingfeng Zhou, Zhanxin Wang, Zhuanqiang Yan, Dingai Wang, Guanzhi Su, Juan Li, Minna Lv, Junjing Hu, Haiming Cai, Yongle Song, Xiangjie Chen, Yibin Zhu, Lijun Yin, Jianfei Zhang, Nanshan Qi, Mingfei Sun

**Affiliations:** ^1^Key Laboratory of Livestock Disease Prevention of Guangdong Province, Key Laboratory of Avian Influenza and Other Major Poultry Diseases Prevention and Control, Ministry of Agriculture and Rural Affairs, Institute of Animal Health, Guangdong Academy of Agricultural Sciences, Guangzhou, China; ^2^Wen’s Group Academy, Wen’s Foodstuffs Group Co., Ltd., Xinxing, Guangdong, China

**Keywords:** broiler chicken, coccidiosis, *Eimeria* spp., prevalence, risk factors

## Abstract

Coccidiosis is a costly intestinal disease of chickens caused by *Eimeria* species. This infection is associated with high mortality, reduced feed efficiency, and slowed body weight gain. The diagnosis and control of coccidiosis becomes challenging due to the fact that chickens can be infected by seven different *Eimeria* species and often occur mixed-species co-infections. Grasping the epidemiology of *Eimeria* species is crucial to estimate the efficiency of poultry management. This study aimed to explore the distribution of *Eimeria* species in broiler chickens in China after administering live anticoccidial vaccines. A total of 634 samples were obtained, and the survey results showed that the prevalence of *Eimeria* was 86.12% (546/634), and the most common species were *E. acervulina* (65.62%), *E. necatrix* (50.95%), *E. mitis* (50.79%), *E. tenella* (48.42%), and *E. praecox* (41.80%). Most samples indicated mixed-species infections (an average of 3.29 species per positive sample). Notably, 63.98% of samples contain 3 to 5 *Eimeria* species within a single fecal sample. The most prevalent combinations were *E. acervulina*–*E. tenella* (38.96%) and *E. acervulina*–*E. necatrix* (37.22%). Statistical analysis showed that flocks vaccinated with trivalent vaccines were significantly positive for *E. necatrix* in grower chickens (OR = 3.30, *p* < 0.05) compared with starter chickens, and tetravalent vaccinated flocks showed that starter chickens demonstrated a higher susceptibility to *E. tenella*–*E. brunetti* (OR = 2.03, *p* < 0.05) and *E. acervulina*–*E. maxima* (OR = 2.05, *p* < 0.05) compared with adult chickens. Geographically, in the case of tetravalent vaccine-immunized flocks, a substantial positive association was observed between *E. necatrix* infection rates and flocks from eastern (OR = 3.88, *p* < 0.001), central (OR = 2.65, *p* = 0.001), and southern China (OR = 3.17, *p* < 0.001) compared with southwestern China. This study also found a positive association between *E. necatrix* (OR = 1.64, *p* < 0.05), *E. acervulina* (OR = 1.59, *p* < 0.05), and *E. praecox* (OR = 1.81, *p* < 0.05) infection and coccidiosis occurrence compared with non-infected flocks in tetravalent vaccinated flocks. This molecular epidemiological investigation showed a high prevalence of *Eimeria* species in the field. The emergent species, *E. brunetti* and *E. praecox*, might be incorporated into the widely-used live vaccines in the future. These insights could be useful in refining coccidiosis control strategies in the poultry industry.

## 1 Introduction

Coccidiosis is a worldwide intestinal disease of chickens caused by protozoan parasites belonging to the genus *Eimeria* (1). Seven *Eimeria* species have been well-known to be responsible for avian coccidiosis (2, 3), and recently, three cryptic species designated as Operational Taxonomic Units (OTUs) X, Y, and Z, have been suggested and assigned by Blake et al. (4). These species of *Eimeria* exhibit varying degree of pathogenicity, with *E. necatrix* standing out as the most virulent species, while *E. tenella* is more prevalent, both inflicting bloody lesions, high morbidity, and high mortality in naïve chickens (5); *E. brunetti* is highly pathogenic and associated with hemorrhagic coccidiosis (6); *E. acervulina*, *E. maxima*, *E. mitis*, and *E. praecox* are usually less pathogenic, incurring malabsorption and enteritis (6). Avian coccidiosis can cause substantial financial losses, with an estimated global cost to the poultry industry approximately £10.36 billion annually (1).

Currently, the primary methods of controlling coccidiosis encompass preventative chemotherapy using in-feed anticoccidial drugs and live vaccines. However, the emergence of drug resistance in various regions worldwide has led to a reduction in the efficacy of anticoccidial agents (7). Additionally, concerns regarding the efficacy of live vaccine have been on the rise (8). In China, three types of live anticoccidial vaccines are available for use (National Veterinary Drug Basic Database Online),^1^ in which two of them are attenuated (namely a trivalent vaccine containing *E. tenella*, *E. acervulina*, and *E. maxima* and a tetravalent vaccine containing *E. tenella*, *E. necatrix*, *E. acervulina*, and *E. maxima*). Another one is non-attenuated, known as Coccivac™ containing *E. maxima*, *E. mivati*, *E. acervulina*, and *E. tenella*. To evaluate the efficiency of poultry management, including the selection of *Eimeria* type in vaccines, a comprehensive understanding of the epidemiology of *Eimeria* species is essential.

Previous reports have monitored the patterns of oocyst accumulation in litter following vaccination (8, 9). The results have revealed a notable increase, marked by a small peak in the reproduction of vaccine strains between 2 and 4 weeks after vaccination at 1 week, followed by another slightly higher peak occurred at 4–7 weeks, which represented a challenge from the local virulent population. Subsequently, oocyst production reduced due to flock immunity after approximately 7 or 8 weeks, with no detectable oocysts remaining in the litter using conventional techniques. Similarly, changes in *Eimeria* oocyst concentration and species composition in litter of broiler farms subjected to various cycles of anticoccidial drug or live *Eimeria* oocyst vaccine control have been reported by Jenkins et al. (9). The influence of anticoccidial methods on the presence and composition of *Eimeria* species in the litter has also been underscored (10). These studies suggest that understanding the species composition in litter during live vaccine control may serve as a means for assessing the efficacy of a particular control program. However, comprehensive data regarding the prevalence and risk factors associated with *Eimeria* infection in broiler chickens administrated with live anticoccidial vaccines in China have been lacking.

Traditional classification of *Eimeria* species depends on morphological characteristics, the affected region of the intestinal tract, and the pre-patent period of *Eimeria* after passage through experimentally infected chickens. However, these methods may not achieve a species-specific diagnosis due to overlap in these characteristics among certain species (11). Recently, polymerase chain reaction (PCR) was used to identify all seven avian *Eimeria* species. These techniques employ genetic markers in the internal transcribed spacer-1 (ITS-1) (12, 13), ITS-2 (14), and sequence characterized amplified region (SCAR) (12). In the present study, we applied quantitative real-time PCR (qPCR) to conduct a comprehensive survey aimed at assessing the distribution of *Eimeria* species in broiler flocks vaccinated with live vaccine across China. This survey was designed to determine the prevalence of *Eimeria* infection and the risk factors in flocks.

## 2 Materials and methods

### 2.1 Sample area and farms

**Figure 1:**
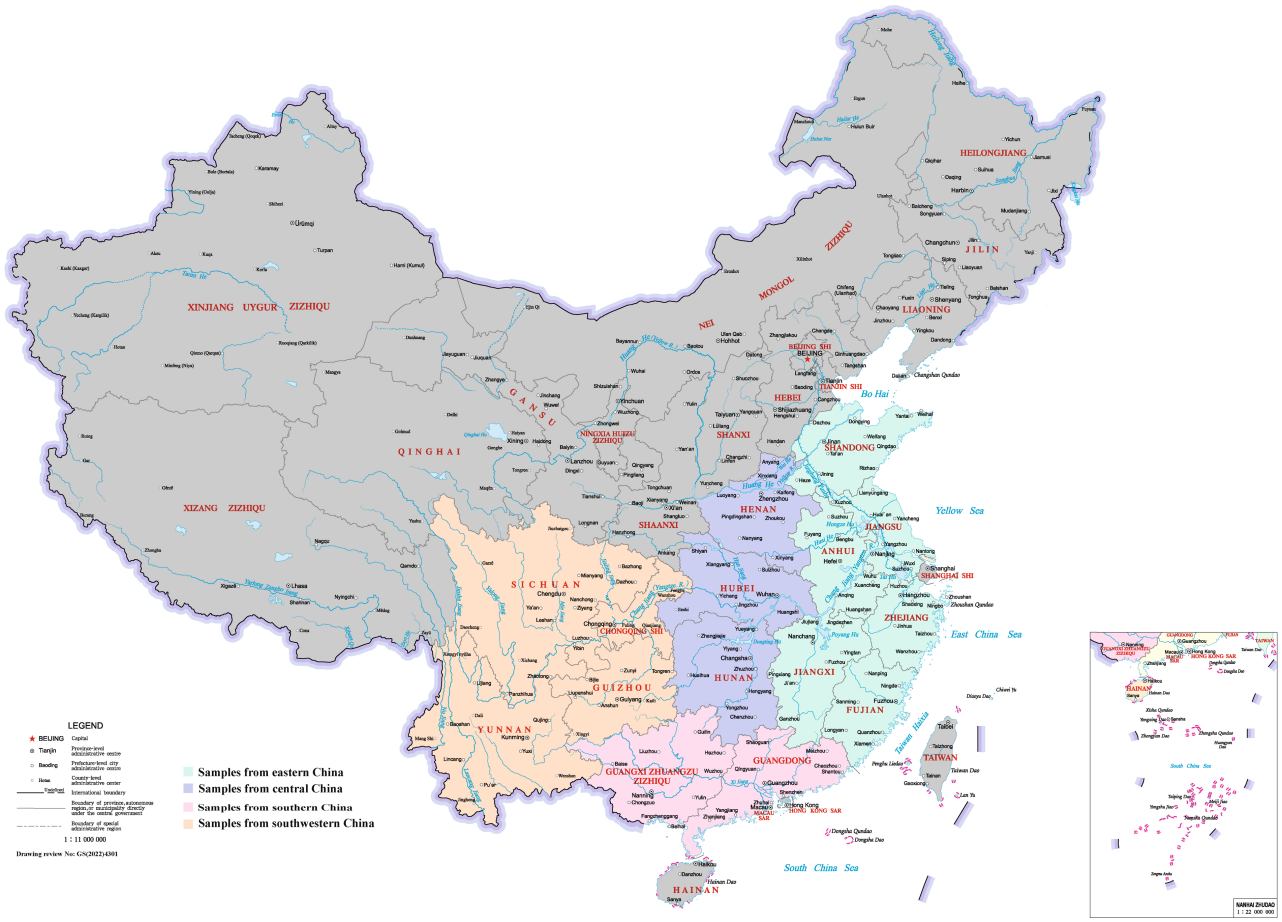
Geographic location of the sampling sites in China. Samples from Eastern, Central, Southern, and Southwestern China are shaded as indicated. This image was obtained from the Ministry of Natural Resources of the People’s Republic of China, with drawing review number [GS(2022)4301]. Reproduced with permission.

The study was conducted from September to November 2022, encompassing a research area that spanned 15 provinces. The region extends between 20°09′–38°24′ north latitude and 97°21′–123°10′ east longitude, located in southern part of China, covering an area of 2,920,300 km^2^. The provinces in the eastern China experienced temperate and subtropical monsoon climates and subtropical humid climates. Central China had temperate and subtropical monsoon climates, while southern China had subtropical monsoon climates characterized by long summer. In southwestern China, the climates varied, encompassing subtropical humid climates, temperate and subtropical monsoon climates, and mountain climates. During the autumn of 2022, the monthly average temperature ranged from 14 to 25°C. The relative humidity levels fluctuated between 62 and 79%, while average rainfall varied approximately from 30.03 mm to 62.50 mm, as reported by World Weather Online.^2^ This study involved 49 broiler farms, with 14 located in eastern China, 6 in central China, 18 in southern China, and 11 in southwestern China (Figure 1). Each farm was equipped with 2–20 houses, housing between 2000 and 11,000 birds at a density ranging from 10 to 14 birds/m^2^. The litter material used was either wood shavings or rice husk. The most common broiler breeds were yellow-feathered chickens, followed by hybrid broilers. All these farms administrated bio-shuttle program to prevent and control coccidiosis. Live anticoccidial vaccines were administrated in the drinking water between 3 and 5 days of age. The trivalent vaccine comprising *E. tenella*, *E. acervulina,* and *E. maxima* or tetravalent vaccine containing *E. tenella*, *E. necatrix*, *E. acervulina,* and *E. maxima* were used in this study. An anticoccidial drug (e.g., nicarbazin, maduramicin, diclazuril, monensin, or narasin) was added to the grower feed to decrease the adverse effects on performance during peak lesion and oocyst shedding.

### 2.2 Study flocks and samples

We collected fecal samples from 9 to 18 flocks per farm. Flocks from each farm were subsequently categorized into three groups according to the age: starter (1–4 weeks), grower (5–8 weeks), and adult (over 8 weeks). Approximately one-third of the samples were selected from each group on each farm. Each individual sample weighed approximately 250 g and was consisted of 30 fresh fecal droppings collected randomly from various locations with each poultry house. A total of 634 fecal samples were collected from 49 farms and placed in labeled zipped plastic bags. All the samples were sent immediately to the laboratory and stored at 4°C for further use.

### 2.3 Genomic DNA extraction from samples

Before extracting DNA, each sample was added to an equal volume of sterile ddH_2_O and was homogenized using a blender. Aliquots of 200 μL were transferred to 1.5 mL Eppendorf tubes for DNA extraction. Genomic DNA extraction was performed using the E.Z.N.A.^®^ Stool DNA Kit, according to the manufacturer’s protocol (Omega, D4015). The resulting DNA was stored at −20°C until further analysis.

### 2.4 Molecular characterization of *Eimeria* species using qPCR

Identification of *Eimeria* species was accomplished via qPCR using species-specific primers (Table 1), as previously described by Vrba et al. (13) and Haug et al. (15), with some modifications. For each sample, the total volume of 20 μL was mixed containing 10 μL of TB Green *Premix Ex Taq* II (Takara, RR820B), 1 μL (100 nm) of species-specific forward and reverse primers, 2 μL of DNA sample, and 6 μL of ultra-pure H_2_O. The amplification process was performed using CFX Connect™ Real-Time PCR System (Bio-Rad, United States), employing a cycling condition that commenced with an initial hold at 95°C for 30 s, followed by 40 repeat cycles of hold at 95°C for 5 s and annealing at 60°C for 30 s. Upon the completion of the cyclic amplification, a melting curve analysis ranging from 65°C to 95°C was determined.

**Table 1:** Quantitative real-time PCR primers for the seven chicken *Eimeria* species.

*Eimeria* species	Primer name	Primer sequences 5′ – 3′	Expected amplicon size (bp)	Melting temperature of amplicon (°C)
*Eimeria necatrix*	NECF^a^	AACGCCGGTATGCCTCGTCG	134	85.0
	NECR^a^	GTACTGGTGCCAACGGAGA		
*Eimeria tenella*	TENF^a^	TCGTCTTTGGCTGGCTATTC	100	86.5
	TENR^a^	CAGAGAGTCGCCGTCACAGT		
*Eimeria brunetti*	BRUF^a^	AGCGTGTAATCTGCTTTTGGAA	118	83.0
	BRUR^a^	TGGTCGCAGACGTATATTAGGG		
*Eimeria acervulina*	ACEF^a^	GCAGTCCGATGAAAGGTATTTG	103	81.5
	ACER^a^	GAAGCGAAATGTTAGGCCATCT		
*Eimeria maxima*	MAXF^a^	TCGTTGCATTCGACAGATTC	138	86.5
	MAXR^a^	TAGCGACTGCTCAAGGGTTT		
*Eimeria mitis*	MITF^a^	CAAGGGGATGCATGGAATATAA	115	82.0
	MITR^a^	CAAGACGAATGGAATCAATCTG		
*Eimeria praecox*	EPF^b^	CATCGGAATGGCTTTTTGAAAGCG	215	82.5
	EPR^b^	GCATGCGCTAACAACTCCCCTT		

a. Primers described by Vrba et al. (13). b. Primers described by Haug et al. (15).

### 2.5 Statistical analysis

All statistical analyses were performed using software IBM SPSS Statistics 27.0 (SPSS Inc.).^3^ Descriptive statistics including bird age, coccidiosis vaccination status, and the presence of clinical signs (e.g., bloody feces, gross lesions of *Eimeria* species) of coccidiosis were obtained from the questionnaires. Initially, the prevalence of *Eimeria* spp. infections with a 95% confidence interval (CI) was calculated. Subsequently, predictor variables associated with the presence of *Eimeria* spp. were assessed using univariable logistic regression models. Chi-square test or Fisher’s exact test was used to compare the prevalence of one or more *Eimeria* infection in variables according to age, vaccination, clinical sign, and region. Odds ratio (OR, with 95% CI) was calculated to assess the associations between participants’ characteristics and *Eimeria* spp. infection. Data with *p* values ≤0.05 were considered as statistical significance.

## 3 Results

### 3.1 *Eimeria* species occurrence

A total of 634 samples were collected from broiler farms across four regions in China (Figure 1). Out of these, 546 (86.12%) from 634 flocks were identified to be positive for one or more *Eimeria* species using species-specific qPCR (Table 2). All seven *Eimeria* species were detected in chickens vaccinated with either trivalent or tetravalent live vaccines with different detection rates, among which *E. acervulina* (65.62%), *E. necatrix* (50.95%), *E. mitis* (50.79%), *E. tenella* (48.42%), and *E. praecox* (41.80%) were the most common species in China. However, the prevalence of *E. maxima* (4.42%) was much lower than the other six *Eimeria* species, which ranged from 21.61 to 65.62%. In flocks that used trivalent vaccines, the distribution of *E. necatrix* in grower flocks (52.38%, *p* < 0.05) was more widespread than those in starter flocks, and *E. tenella* in starter flocks (53.13%, *p* < 0.05) was more widespread than those in adult flocks. In flocks that used tetravalent vaccines, the prevalence of *E. necatrix* was significantly higher in starter flocks (66.36%, *p* = 0.001) compared with grower flocks, as well as the prevalence of *E. tenella* in both starter flocks (62.62%, *p* < 0.05) and grower flocks (50.44%, *p* < 0.05) was significantly higher compared with adult flocks.

**Table 2:** Prevalence of *Eimeria* infection in broiler Chickens from China.

*Eimeria* species	All (*n* = 634)	Trivalent vaccine (*n* = 112)				Tetravalent vaccine (*n* = 522)			
	Positive (95% CI)	Starter (*n* = 32) positive (95% CI)	Grower (*n* = 63) positive (95% CI)	Adult (*n* = 17) positive (95% CI)	*p*-value	Starter (*n* = 107) positive (95% CI)	Grower (*n* = 343) positive (95% CI)	Adult (*n* = 72) positive (95% CI)	*p*-value
Any *Eimeria* species	86.12 (83.42–88.82)	93.75 (84.88–100)	**96.83 (92.37–100)**	76.47 (53.99–98.95)	**0.015**	89.72 (83.87–95.57)	84.26 (80.38–88.13)	79.17 (69.56–88.78)	0.147
*Eimeria necatrix*	50.95 (47.04–54.85)	25.0 (9.14–40.86)	**52.38 (39.70–65.06)**	47.06 (20.61–73.51)	**0.038**	**66.36 (57.26–75.45)**	46.94 (41.63–52.25)	58.33 (46.67–70.0)	**0.001**
*Eimeria tenella*	48.42 (44.52–52.32)	**53.13 (34.85–71.40)**	34.92 (22.82–47.02)	11.76 (0–28.84)	**0.015**	**62.62 (53.30–71.93)**	**50.44 (45.12–55.76)**	36.11 (24.74–47.48)	**0.002**
*Eimeria brunetti*	21.61 (18.40–24.82)	12.50 (0.39–24.61)	26.98 (15.72–38.25)	17.65 (0–37.85)	0.245	15.89 (8.85–22.93)	23.32 (18.83–27.82)	22.22 (12.38–32.06)	0.263
*Eimeria acervulina*	65.62 (61.91–69.32)	78.13 (62.98–93.27)	79.37 (69.09–89.64)	52.94 (26.49–79.39)	0.074	70.09 (61.28–78.91)	63.56 (58.44–68.68)	54.17 (42.38–65.96)	0.095
*Eimeria maxima*	4.42 (2.81–6.02)	12.5 (0.39–24.61)	3.17 (0–7.63)	11.76 (0–28.84)	0.176	**8.41 (3.07–13.76)**	2.62 (0.92–4.32)	2.78 (0–6.67)	**0.042**
*Eimeria mitis*	50.79 (46.89–54.69)	78.13 (62.98–93.27)	77.78 (67.22–88.33)	52.94 (26.49–79.39)	0.096	39.25 (29.85–48.66)	**51.31 (46.0–56.63)**	29.17 (18.41–39.92)	**<0.001**
*Eimeria praecox*	41.80 (37.95–45.65)	50.0 (31.68–68.32)	49.21 (36.51–61.90)	29.41 (5.26–53.56)	0.311	36.45 (27.18–45.72)	42.86 (37.59–48.12)	37.50 (26.04–48.96)	0.414

n, total number of samples; 95% CI: 95% confidence interval; significant predictors in bold.

**Figure 2:**
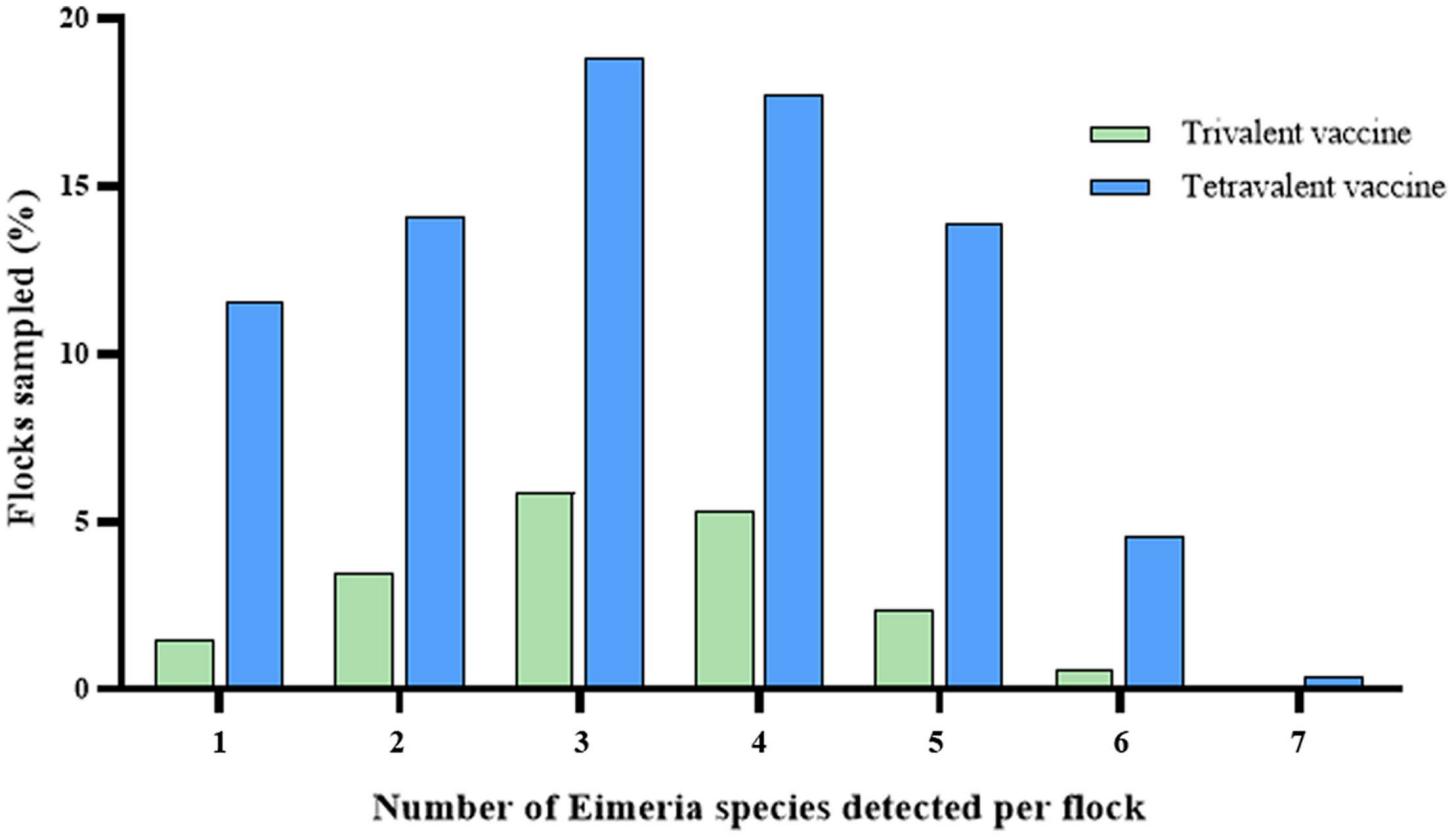
Frequency of mixed infections in broiler chickens from China.

Mixed-species infections were common in China, with an average of 3.29 species per positive sample. Notably, 63.98% of samples contained 3 to 5 *Eimeria* species within a single fecal sample (Figure 2). The prevalence of mixed infections with three species was higher in both trivalent vaccine-immunized flocks (5.85%) and tetravalent vaccine-immunized flocks (18.83%; Table 3). The most prevalent combinations *Eimeria* species included *E. acervulina*–*E. tenella* (38.96%), *E. acervulina*–*E. necatrix* (37.22%), and *E. acervulina*–*E. tenella*–*E. necatrix* (25.08%). Specifically, the prevalence of *E. acervulina*–*E. necatrix* was significantly higher in grower flocks (38.10%, *p* < 0.05) compared with starter flocks within trivalent vaccine-immunized flocks. Furthermore, the prevalence of *E. acervulina*–*E. necatrix* (52.34%, *p* < 0.05), *E. acervulina*–*E. tenella* (50.47%, *p* < 0.05), and *E. acervulina*–*E. tenella*–*E. necatrix* (41.12%, *p* < 0.05) was significantly higher in starter flocks compared with adult flocks in tetravalent vaccine-immunized flocks.

**Table 3:** Prevalence of mixed infections in broiler chickens from China.

*Eimeria* species	All (*n* = 586)	Trivalent vaccine				Tetravalent vaccine			
	Positive (95% CI)	Starter (*n* = 32) positive (95% CI)	Grower (*n* = 63) positive (95% CI)	Adult (*n* = 17) positive (95% CI)	*p*-value	Starter (*n* = 107) positive (95% CI)	Grower (*n* = 343) positive (95% CI)	Adult (*n* = 72) positive (95% CI)	*p*-value
EA–ET	38.96 (35.65–42.77)	40.63 (22.63–58.62)	31.75 (19.93–43.56)	11.76 (0–28.84)	0.115	**50.47 (40.84–60.10)**	39.94 (34.73–45.15)	29.17 (18.41–39.92)	**0.016**
EA–EN	37.22 (33.45–41.0)	12.50 (0.39–24.61)	**38.10 (25.77–50.42)**	29.41 (5.26–53.56)	**0.035**	**52.34 (42.72–61.95)**	35.86 (30.76–40.96)	33.33 (22.18–44.49)	**0.006**
EA–ET–EN	25.08 (21.70–28.46)	6.25 (0–15.12)	17.46 (7.82–27.10)	11.76 (0–28.84)	0.310	**41.12 (31.65–50.60)**	24.78 (20.19–29.37)	20.83 (11.22–30.44)	**0.002**
EA–ET–EMI	26.50 (23.05–29.94)	37.50 (19.77–55.23)	26.98 (15.72–38.25)	11.76 (0–28.84)	0.157	25.23 (16.87–33.60)	28.86 (24.04–33.68)	15.28 (6.76–23.79)	0.057
EA–ET–EB	11.99 (9.45–14.52)	6.25 (0–15.12)	9.52 (2.07–16.98)	0	0.218	10.28 (4.43–16.13)	14.87 (11.08–18.65)	8.33 (1.79–14.87)	0.208
EA–ET–EN–EMI	16.56 (13.66–19.46)	6.25 (0–15.12)	15.87 (6.60–25.15)	11.76 (0–28.84)	0.370	18.69 (11.18–26.20)	17.78 (13.72–21.85)	13.89 (5.71–22.07)	0.679
EA–ET–EN–EB	7.89 (5.78–9.99)	3.13 (0–9.50)	6.35 (0.16–12.54)	0	0.338	7.48 (2.41–12.54)	9.33 (6.24–12.42)	6.94 (0.93–12.96)	0.721

n, total number of samples; 95% CI: 95% confidence interval; significant predictors in bold. Eimeria species: E. acervulina (EA), E. tenella (ET), E. necatrix (EN), E. mitis (EMI), E. brunetti (EB).

### 3.2 Risk factors associated with *Eimeria* species occurrence

Odds ratio associated with the likelihood of occurrence of *Eimeria* was calculated based on bird age, presence of clinical sign, and sample region (Table 4). The results of the statistical analysis reveal significant associations between the type of vaccines used and the prevalence of specific *Eimeria* species. In flocks vaccinated with trivalent vaccines, a significant positive association was observed for *E. necatrix* infection in grower chickens (OR = 3.30, 95% CI: 1.29–8.45; *p* < 0.05) compared with starter chickens, and these flocks exhibited a significant positive association with *E. mitis*–*E. praecox* infection in both starter chickens (OR = 3.85, 95% CI: 1.05–14.16; *p* < 0.05) and grower chickens (OR = 4.71, 95% CI: 1.47–15.15; *p* < 0.05) compared with adult chickens. In the case of tetravalent vaccinated flocks, starter chickens were more likely to be positive for *E. tenella*–*E. brunetti* (OR = 2.03, 95% CI: 1.10–3.73; *p* < 0.05) and *E. acervulina*–*E. maxima* (OR = 2.05, 95% CI: 1.10–3.85; *p* < 0.05) compared with adult chickens, followed by a higher likelihood of positive results for *E. mitis*–*E. praecox* infection in grower chickens (OR = 2.0, 95% CI: 1.20–3.34; *p* < 0.05) compared with adult chickens.

**Table 4:** Univariable logistic regression analysis of risk factors associated with prevalence of *Eimeria* species in broiler chickens in China.

Groups	Variables	Category	*E. necatrix*			*E. tenella*–*E. brunetti*			*E. acervulina*–*E. maxima*			*E. mitis*–*E. praecox*		
			Positive (95% CI)	OR (95% CI)	*p*-value	Positive (95% CI)	OR (95% CI)	*p*-value	Positive (95% CI)	OR (95% CI)	*p*-value	Positive (95% CI)	OR (95% CI)	*p*-value
Trivalent vaccine	Age	Starter (*n* = 32)	25.0 (9.14–40.86)	Referent	—	59.38 (41.38–77.37)	3.51 (1.0–12.36)	0.051	81.25 (66.95–95.55)	3.03 (0.82–11.26)	0.097	81.25 (66.95–95.55)	**3.85 (1.05–14.16)**	**0.042**
		Grower (*n* = 63)	52.38 (39.70–65.06)	**3.30 (1.29–8.45)**	**0.013**	47.62 (34.94–60.30)	2.18 (0.69–6.92)	0.185	80.95 (70.98–90.92)	2.98 (0.94–9.42)	0.064	84.13 (74.85–93.40)	**4.71 (1.47–15.15)**	**0.009**
		Adult (*n* = 17)	47.06 (20.61–73.51)	2.67 (0.77–9.25)	0.122	29.41 (5.26–53.56)	Referent	—	58.82 (32.74–84.91)	Referent	—	52.94 (26.49–79.39)	Referent	—
	Region	Eastern (*n* = 36)	47.22 (30.09–64.35)	1.59 (0.56–4.53)	0.385	30.56 (14.75–46.36)	Referent	—	77.78 (63.51–92.04)	Referent	—	80.56 (66.97–94.14)	Referent	—
		Central (*n* = 0)	—	—	—	—	—	—	—	—	—	—	—	—
		Southern (*n* = 51)	45.10 (30.96–59.23)	1.46 (0.55–3.91)	0.451	56.86 (42.79–70.93)	**3.0 (1.22–7.37)**	**0.017**	74.51 (62.13–86.89)	0.84 (0.31–2.29)	0.726	76.47 (64.42–88.52)	0.78 (0.28–2.24)	0.650
		Southwestern (*n* = 25)	36.0 (15.78–56.22)	Referent	—	56.0 (35.09–76.91)	**2.89 (1.0–8.36)**	**0.050**	84.0 (68.56–99.44)	1.50 (0.40–5.65)	0.549	80.01 (63.15–96.85)	0.97 (0.27–3.48)	0.957
	Clinic signs	No (*n* = 75)	38.67 (27.39–49.95)	Referent	—	52.01 (40.43–63.57)	Referent	—	80.0 (70.73–89.27)	Referent	—	74.67 (64.59–84.87)	Referent	—
		Yes (*n* = 37)	54.05 (37.21–70.90)	1.87 (0.84–4.14)	0.125	40.54 (23.95–57.14)	0.63 (0.28–1.40)	0.255	72.97 (57.96–87.98)	0.68 (0.27–1.69)	0.402	86.49 (74.93–98.04)	2.17 (0.74–6.37)	0.158
Tetravalent vaccine	Age	Starter (*n* = 107)	66.36 (57.26–75.45)	1.41 (0.76–2.61)	0.276	64.49 (55.27–73.70)	**2.03 (1.10–3.73)**	**0.023**	71.96 (63.31–80.61)	**2.05 (1.10–3.85)**	**0.025**	53.27 (43.66–62.88)	1.43 (0.78–2.60)	0.247
		Grower (*n* = 343)	46.94 (41.63–52.25)	0.63 (0.38–1.06)	0.080	55.69 (50.40–60.97)	1.40 (0.84–2.34)	0.191	64.14 (59.04–69.24)	1.43 (0.86–2.39)	0.712	61.52 (56.34–66.69)	**2.0 (1.20–3.34)**	**0.008**
		Adult (*n* = 72)	58.33 (46.67–70.0)	Referent	—	47.22 (35.41–59.04)	Referent	—	55.56 (43.80–67.31)	Referent	—	44.44 (32.69–56.20)	Referent	—
	Region	Eastern (*n* = 149)	62.42 (54.55–70.28)	**3.88 (2.29–6.55)**	**<0.001**	53.69 (45.59–61.79)	1.16 (0.71–1.90)	0.557	75.84 (68.89–82.79)	**2.26 (1.32–3.85)**	**0.003**	63.76 (55.95–71.57)	**1.96 (1.19–3.24)**	**0.008**
		Central (*n* = 79)	53.16 (41.92–64.41)	**2.65 (1.45–4.83)**	**0.001**	65.82 (55.13–76.51)	**1.93 (1.06–3.50)**	**0.031**	69.62 (59.25–79.99)	1.65 (0.89–3.04)	0.109	68.35 (57.87–78.84)	**2.41 (1.32–4.41)**	**0.004**
		Southern (*n* = 184)	57.61 (50.40–64.82)	**3.17 (1.92–5.24)**	**<0.001**	58.15 (50.96–65.35)	1.39 (0.86–2.23)	0.174	57.07 (49.85–64.28)	0.96 (0.59–1.54)	0.851	53.80 (46.53–61.08)	1.30 (0.81–2.09)	0.279
		Southwestern (*n* = 110)	30.0 (21.30–38.70)	Referent	—	50.0 (40.51–59.49)	Referent	—	58.18 (48.82–67.55)	Referent	—	47.27 (37.80–56.75)	Referent	—
	Clinic signs	No (*n* = 409)	49.88 (45.01–54.74)	Referent	—	52.81 (47.95–57.67)	Referent	—	62.35 (57.63–67.06)	Referent	—	54.28 (49.43–59.13)	Referent	—
		Yes (*n* = 113)	61.95 (52.86–71.04)	**1.64 (1.07–2.51)**	**0.024**	69.03 (60.37–77.68)	**1.99 (1.28–3.10)**	**0.002**	72.57 (64.21–80.92)	**1.60 (1.01–2.53)**	**0.046**	69.03 (60.37–77.68)	**1.88 (1.21–2.93)**	**0.005**

n, total number of samples; 95% CI: 95% confidence interval; OR: odds ratio; significant predictors in bold.

Geographically, a significant positive association was identified between *E. tenella*–*E. brunetti* infection rate and flocks vaccinated with trivalent vaccine in southern China (OR = 3.0, 95% CI: 1.22–7.37; *p* < 0.05) and southwestern China (OR = 2.89, 95% CI: 1.0–8.36; *p* = 0.05) compared with eastern China (Table 4). In the case of tetravalent vaccine-vaccinated flocks, there was a significant positive association between *E. necatrix* infection and flocks from eastern China (OR = 3.88, 95% CI: 2.29–6.55; *p* < 0.001), central China (OR = 2.65, 95% CI: 1.45–4.83; *p* = 0.001), and southern China (OR = 3.17, 95% CI: 1.92–5.24; *p* < 0.001) compared with southwestern China. Similarly, the tetravalent vaccinated flocks in central China were more likely to be positive for *E. tenella*–*E. brunetti* (OR = 1.93, 95% CI: 1.06–3.50; *p* < 0.05) compared with southwestern China. Interestingly, in flocks vaccinated with tetravalent vaccines, there was a significant positive association between the occurrence of clinical coccidiosis and the prevalence of *E. necatrix* (OR = 1.64, 95% CI: 1.07–2.51; *p* < 0.05), *E. tenella*–*E. brunetti* (OR = 1.99, 95% CI: 1.28–3.10; *p* < 0.05), *E. acervulina*–*E. maxima* (OR = 1.60, 95% CI: 1.01–2.53; *p* < 0.05), and *E. mitis*–*E. praecox* (OR = 1.88, 95% CI: 1.21–2.93; *p* < 0.05) compared with flocks without clinical signs.

### 3.3 *Eimeria* species profiles associated with coccidiosis occurrence

Univariable logistic regression analysis was performed to identify *Eimeria* profiles associated with trivalent vaccines or tetravalent vaccines used strategies in broiler flocks. In flocks that used trivalent vaccines, those infected with *E. maxima* were more likely to develop clinical coccidiosis compared with non-infected flocks (OR = 7.07, 95% CI: 1.35–36.95; *p* < 0.05; Table 5). Furthermore, in flocks that used tetravalent vaccines, those infected with *E. necatrix* (OR = 1.64, 95% CI: 1.07–2.51; *p* < 0.05), *E. acervulina* (OR = 1.59, 95% CI: 1.01–2.51; *p* < 0.05), and *E. praecox* (OR = 1.81, 95% CI: 1.19–2.75; *p* < 0.05) were more likely to occur clinical coccidiosis compared with non-infected flocks. However, no significant associations were observed between the infection of *E. tenella, E. brunetti,* and *E. mitis* and the occurrence of clinical coccidiosis in both trivalent vaccines and tetravalent vaccines used in flocks.

**Table 5:** Univariable logistic regression analysis of risk factors associated with *Eimeria* species presence and coccidiosis occurrence in broiler chickens in China.

Variables	Category	Trivalent vaccine				Tetravalent vaccine			
		No. tested	Positive (95% CI)	OR (95% CI)	*p*-value	No. tested	Positive (95% CI)	OR (95% CI)	*p*-value
*Eimeria necatrix*	No	63	26.98 (15.72–38.25)	Referent	—	248	17.34 (12.59–22.08)	Referent	—
	Yes	49	40.82 (26.56–55.08)	1.87 (0.84–4.14)	0.125	274	25.55 (20.35–30.74)	**1.64 (1.07–2.51)**	**0.024**
*Eimeria tenella*	No	71	35.21 (23.83–46.60)	Referent	—	256	18.75 (13.94–23.56)	Referent	—
	Yes	41	29.27 (14.73–43.81)	0.76 (0.33–1.75)	0.520	266	24.44 (19.24–29.63)	1.40 (0.92–2.13)	0.116
*Eimeria brunetti*	No	88	35.23 (25.05–45.41)	Referent	—	409	19.80 (15.93–23.68)	Referent	—
	Yes	24	25.0 (6.32–43.68)	0.61 (0.22–1.70)	0.348	113	28.32 (19.88–36.75)	1.60 (0.99–2.58)	0.053
*Eimeria acervulina*	No	28	46.43 (26.74–66.12)	Referent	—	190	16.84 (11.47–22.21)	Referent	—
	Yes	84	28.57 (18.71–38.43)	0.46 (0.19–1.11)	0.085	332	24.40 (19.75–29.04)	**1.59 (1.01–2.51)**	**0.045**
*Eimeria maxima*	No	104	29.81 (20.87–38.75)	Referent	—	502	21.31 (17.72–24.91)	Referent	—
	Yes	8	75.0 (36.30–100)	**7.07 (1.35–36.95)**	**0.021**	20	30.0 (8.0–52.0)	1.58 (0.59–4.22)	0.359
*Eimeria mitis*	No	29	27.59 (10.28–44.89)	Referent	—	283	20.14 (15.44–24.84)	Referent	—
	Yes	83	34.94 (24.47–45.41)	1.41 (0.56–3.58)	0.470	239	23.43 (18.02–28.84)	1.21 (0.80–1.84)	0.364
*Eimeria praecox*	No	60	30.0 (18.06–41.94)	Referent	—	309	17.48 (13.22–21.73)	Referent	—
	Yes	52	36.54 (23.0–50.08)	1.34 (0.61–2.96)	0.464	213	27.70 (21.64–33.76)	**1.81 (1.19–2.75)**	**0.006**

95% CI: 95% confidence interval; OR: odds ratio; significant predictors in bold.

## 4 Discussion

Coccidiosis is a disease of significant economic concern worldwide in the poultry industry. The epidemiological monitoring of *Eimeria* species present in broiler chickens which are vaccinated with live vaccines is vital for the selection of prevention and controlling strategies for coccidiosis. The investigation in our study included seven well-known *Eimeria* species, excluding the three cryptic species OTU_X_, OTU_Y_, and OTU_Z_. This survey was conducted based on the fact that the main prevalence species in China are the seven known species (16–19). Additionally, there have been few studies reporting the presence of unidentifiable OTUs in chickens immunized with transgenic *Eimeria* in China (20). The overall prevalence of coccidiosis in China is 86.12% (546 of 634 flocks). A comparably high prevalence has been reported in provinces in China such as Anhui (87.75%) (18), Hubei (97.79%), and Henan (96.70%) (16) and other countries including Greece (85.7%) (21), northeastern Algeria (99.5%) (22), Colombia (96.3%) (23), and Australia (98%) (24). Conversely, the prevalence was notably lower in Serbia, north India, Korean, northeastern Brazil, and southwestern Nigeria with rates of 59, 28.5, 75, 59, and 41.3% (25–29), respectively. The variance in *Eimeria* species occurrence can be attributed to differences in control methods, sampling times, animal management practices, and climatic conditions (30). Previous monitoring trials found that flocks fed with anticoccidial agent in diet produced a considerably higher count of the oocysts in vaccinated flocks, especially between 4 and 8 weeks (8). Consistently, our study also found that the prevalence in vaccinated flocks (86.12%) was lower than in flocks administered with anticoccidial agent (97.17%), as previously reported (16). Southern China’s warm and humid autumn climates, with an average temperature of 25°C and a relative humidity of 79%, may contribute to the elevated prevalence of *Eimeria* in broiler flocks. However, it is noteworthy that we did not find an increased number of *Eimeria*-positive flocks in southern China compared with the other three regions in China, suggesting more effective control measures being implemented in those areas.

Seven species of *Eimeria* were populated in broiler chicken farms across China. The most prevalent species included *E. acervulina* (65.62%), *E. necatrix* (50.95%), *E. mitis* (50.79%), and *E. tenella* (48.42%). Interactions among *Eimeria* species, coupled with crowding effect, are known as the most important factors affecting oocyst production (31). Notably, *E. acervulina* and *E. tenella* exhibit a higher reproductive potential, and in co-infections, *E. acervulina* reduces the oocyst production of *E. necatrix*, *E. maxima*, and *E. brunetti* (31). The mixed infection of *E. acervulina*–*E. tenella* (38.96%) and *E. acervulina*–*E. necatrix* (37.22%) was the dominant co-infection in our study. Interestingly, younger flocks vaccinated with tetravalent vaccines and aged younger than 4 weeks showed a higher likelihood of detecting positive for *E. acervulina*–*E. tenella* (50.47%, *p* < 0.05), *E. acervulina*–*E. necatrix* (52.34%, *p* < 0.05), and *E. acervulina*–*E. tenella*–*E. necatrix* (41.12%, *p* < 0.05) compared with older ones. This is in agreement with previous studies indicating that found younger chickens are more susceptible to *Eimeria* species (8, 30, 32). An exception was observed with *E. maxima*, in which the positive rate was extremely low (4.42%). This might be attributed to all the studied flocks being vaccinated with *E. tenella, E. acervulina, and E. maxima* or *E. tenella, E. necatrix, E. acervulina, and E. maxima.* One possible explanation for this could be that the primers for *E. maxima* failed to detect all strains present in the field samples, as demonstrated by Lew et al. (33) and Sun et al. (34). Alternatively, another reason may be some associated factors affecting the reproductive potential of the live *E. maxima* vaccine strains, since the positive rate of *E. maxima* was not higher in starter chickens compared with grower and adult chickens, especially in trivalent vaccines used flocks. This is inconsistent with the finding reported by Snyder et al. (35) that vaccinated flocks had peak oocyst shedding during weeks 2 to 4. Since the intestinal lesions caused by coccidiosis (especially *E. maxima*) were a well-known predisposing factor for necrotic enteritis (NE). The prevention of coccidiosis and NE was intimately connected due to the risk of NE was greater if the number of oocysts is excessive for *E. maxima*. The low frequency of *E. maxima* in this study might reduce the occurrence of NE in live vaccines used flocks. Nevertheless, it is worth noting that the relatively high prevalence of *E. brunetti* peaked at 21.61% in broiler chickens compared with the previous finding (6.6%) in China (16). Given its increased prevalence, *E. brunetti* may need to be included in the vaccine in China, echoing similar recommendation made in Australia as previous report (36).

Different *Eimeria* species distribution, control strategies, and geographical features could affect the occurrence of avian coccidiosis. *E. necatrix* is known to have lower fecundity and to be a ‘poor competitor’ compared with other species (8). However, In the case of tetravalent vaccinated flocks, the prevalence of *E. necatrix* was the highest in starter flocks (66.36%) while decreased in grower flocks (46.94%), followed by a slight increase in adult flocks (58.33%). Furthermore, there was a significant positive association between *E. necatrix* infection rate and coccidiosis occurrence (OR = 1.64, 95% CI: 1.07–2.51; *p* < 0.05), hinting that flocks might challenge by wild-type strains. This association was consistent with the finding identifying *E. necatrix* as a contributing factor to coccidiosis occurrence (8, 35). This finding suggested that factors related to the host (e.g., underlying subclinical non-parasitic infections) and environmental factors (e.g., crowding, air quality, and stress) may negatively affect the health of vaccinated chickens, which increased the susceptibility of chickens to coccidiosis, similar to previous reports (21, 29, 36). Moreover, our study found that flocks infected with *E. acervulina* were also at a significantly higher risk of coccidiosis (OR = 1.59, 95% CI: 1.01–2.51; *p* < 0.05) when vaccinated with tetravalent vaccines. Known for its rapid reproduction and short life cycle, *E. acervulina*’s preponderance in broiler farms and its link to coccidiosis outbreaks resonates with previous study (7). However, flocks vaccinated with trivalent vaccines and infected with *E. maxima* showed a significantly elevated risk of coccidiosis (OR = 7.07, 95% CI: 1.35–36.95; *p* < 0.05). This may be due to the vaccine strain of *E. maxima* that induced incomplete cross-strain immune protection against other wild-type strains (37). Surprisingly, flocks positive for *E. praecox* in tetravalent vaccine-immunized farms were also at an increased risk of coccidiosis (OR = 1.59, 95% CI: 1.02–2.47; *p* < 0.05). Despite often being overlooked, in our study, the high prevalence of *E. praecox* (41.80%) was consistent with previous survey in Henan and Hubei provinces in China (33.33%), Brazil (25.1%), and Australia (34.4%) (16, 24, 28). The pathogenicity of *E. praecox* was observed by Williams et al. for the first time to cause decrease in weight gain and increase in feed conversion ratio (38). Therefore, the high frequencies and risk associated with *E. necatrix*, *E. acervulina*, and *E. praecox* in broilers emphasize the importance of incorporating them into a comprehensive broiler vaccine.

## 5 Conclusion

In summary, suboptimal post-immunization poultry practices, following the administration of live attenuated vaccines, precipitate either isolated or combined infections of *E. acervulina, E. necatrix*, *E. tenella*, *E. mitis*, and *E. praecox*, whether as isolated or combined infections, leading to a pronounced susceptibility to morbidity. Furthermore, trivalent or tetravalent attenuated live vaccines are available in China. It is worth noting that the presence of new dominated *E. brunetti* and *E. praecox* could be included in the widely used live vaccines. Further investigations are needed to evaluate the epidemiology of virulent species in clinical contexts and discern their associated morbidity implications. By formulating an evidence-based immunization strategy and enhancing post-immunization poultry practices, we can regulate the prevalence of clinical coccidian taxa and assiduously curtail their affiliated pathological ramifications. Notably, our study is the first to report the prevalence of *Eimeria* species in flocks vaccinated with live vaccines in China, based on molecular analysis.

## Data availability statement

The original contributions presented in the study are included in the article/Supplementary material, further inquiries can be directed to the corresponding authors.

## Ethics statement

The animal study was approved by the Animal Care and Use Committee of the Institute of Animal Health, Guangdong Academy of Agricultural Sciences. The study was conducted in accordance with the local legislation and institutional requirements.

## Author contributions

SL: Conceptualization, Data curation, Investigation, Software, Writing – original draft. XL: Conceptualization, Data curation, Investigation, Writing – original draft. QZ: Conceptualization, Investigation, Methodology, Writing – original draft. ZW: Investigation, Methodology, Writing – original draft. ZY: Investigation, Methodology, Writing – original draft. DW: Investigation, Methodology, Writing – original draft. GS: Investigation, Methodology, Writing – original draft. JL: Data curation, Methodology, Writing – original draft. ML: Data curation, Methodology, Writing – original draft. JH: Data curation, Methodology, Writing – original draft. HC: Data curation, Methodology, Writing – original draft. YS: Data curation, Methodology, Writing – original draft. XC: Data curation, Methodology, Writing – original draft. YZ: Data curation, Methodology, Writing – original draft. LY: Data curation, Methodology, Writing – original draft. JZ: Methodology, Resources, Supervision, Writing – original draft. NQ: Funding acquisition, Methodology, Project administration, Supervision, Writing – review & editing. MS: Funding acquisition, Methodology, Project administration, Supervision, Writing – review & editing.

## Footnotes

^1^http://124.126.15.169:8081/cx/

^2^https://www.worldweatheronline.com/

^3^http://www.spss.com.hk
