# Supplementary material for: Epidemiological investigation of coccidiosis and associated risk factors in broiler chickens immunized with live anticoccidial vaccines in China
Source: Front Vet Sci. 2024 Mar 19;11:1375026. doi: 10.3389/fvets.2024.1375026 (PMC10986636; doi:10.3389/fvets.2024.1375026)
Supplement: Supplementary file 1 [file Table_1.DOCX]

**Supplementary Data 1**:

**Table S1.** The results of the identification of *Eimeria* species in east China.

| **District** | **Age (days)** | **Vaccine** | **Clinic** | **Coccidia species** |
| --- | --- | --- | --- | --- |
| Yancheng1-1 | 29 | tetravalent vaccine | N | A B N T Mi P |
| Yancheng1-2 | 35 | tetravalent vaccine | N | A |
| Yancheng1-3 | 52 | tetravalent vaccine | N | A N P |
| Yancheng1-4 | 68 | tetravalent vaccine | N | A B T |
| Yancheng1-5 | 25 | tetravalent vaccine | N | A B N T Ma Mi P |
| Yancheng1-6 | 79 | tetravalent vaccine | N | A T P |
| Yancheng1-7 | 44 | tetravalent vaccine | N | nd |
| Yancheng1-8 | 49 | tetravalent vaccine | N | A B N T Mi |
| Yancheng1-9 | 24 | tetravalent vaccine | N | A B N T Mi P |
| Yancheng1-10 | 69 | tetravalent vaccine | N | A N T |
| Yancheng1-11 | 36 | tetravalent vaccine | N | A N Mi P |
| Yancheng1-12 | 38 | tetravalent vaccine | N | nd |
| Nantong1-1 | 38 | tetravalent vaccine | N | nd |
| Nantong1-2 | 32 | tetravalent vaccine | N | N Mi |
| Nantong1-3 | 44 | tetravalent vaccine | N | A |
| Nantong1-4 | 43 | tetravalent vaccine | N | nd |
| Nantong1-5 | 47 | tetravalent vaccine | N | A N Mi P |
| Nantong1-6 | 48 | tetravalent vaccine | N | A Mi P |
| Nantong1-7 | 51 | tetravalent vaccine | N | A N T P |
| Nantong1-8 | 75 | tetravalent vaccine | Y | B N |
| Nantong1-9 | 46 | tetravalent vaccine | N | A |
| Nantong1-10 | 37 | tetravalent vaccine | N | nd |
| Nantong1-11 | 26 | tetravalent vaccine | N | A N T Mi P |
| Nantong1-12 | 21 | tetravalent vaccine | N | A N T Mi P |
| Nantong1-13 | 25 | tetravalent vaccine | N | A N Mi |
| Nantong1-14 | 64 | tetravalent vaccine | Y | N |
| Nantong1-15 | 94 | tetravalent vaccine | Y | A N P |
| Huaian1-1 | 45 | tetravalent vaccine | N | A B N T Mi P |
| Huaian1-2 | 45 | tetravalent vaccine | N | N T |
| Huaian1-3 | 45 | tetravalent vaccine | Y | A N P |
| Huaian1-4 | 166 | tetravalent vaccine | N | N Mi P |
| Huaian1-5 | 88 | tetravalent vaccine | N | N T Ma Mi P |
| Huaian1-6 | 63 | tetravalent vaccine | N | A |
| Huaian1-7 | 40 | tetravalent vaccine | N | A N Mi |
| Huaian1-8 | 33 | tetravalent vaccine | N | A |
| Huaian1-9 | 33 | tetravalent vaccine | Y | A |
| Huaian1-10 | 24 | tetravalent vaccine | N | A N |
| Huaian1-11 | 24 | tetravalent vaccine | Y | A |
| Huaian1-12 | 39 | tetravalent vaccine | N | N |
| Huaian1-13 | 24 | tetravalent vaccine | Y | A N T |

to be continued

| **District** | **Age (days)** | **Vaccine** | **Clinic** | **Coccidia species** |
| --- | --- | --- | --- | --- |
| Huaian1-14 | 82 | tetravalent vaccine | N | A N |
| Huaian1-15 | 58 | tetravalent vaccine | N | A N |
| Nanjing1-1 | 23 | trivalent vaccine | N | N T Mi |
| Nanjing1-2 | 51 | trivalent vaccine | N | A N Mi P |
| Nanjing1-3 | 47 | trivalent vaccine | N | A N Mi P |
| Nanjing1-4 | 49 | trivalent vaccine | Y | Mi P |
| Nanjing1-5 | 25 | trivalent vaccine | N | N Mi |
| Nanjing1-6 | 46 | trivalent vaccine | N | A |
| Nanjing1-7 | 36 | trivalent vaccine | N | A Mi P |
| Nanjing1-8 | 40 | trivalent vaccine | N | A Mi |
| Nanjing1-9 | 33 | trivalent vaccine | N | A Mi P |
| Nanjing1-10 | 44 | trivalent vaccine | N | A Mi P |
| Nanjing1-11 | 27 | trivalent vaccine | N | A N Mi P |
| Nanjing1-12 | 48 | trivalent vaccine | Y | A N P |
| Zhenjiang1-1 | 23 | tetravalent vaccine | N | A N T P |
| Zhenjiang1-2 | 26 | tetravalent vaccine | N | A B N Mi P |
| Zhenjiang1-3 | 29 | tetravalent vaccine | N | A Mi P |
| Zhenjiang1-4 | 29 | tetravalent vaccine | N | A B T Mi P |
| Zhenjiang1-5 | 35 | tetravalent vaccine | N | A N |
| Zhenjiang1-6 | 39 | tetravalent vaccine | N | A P |
| Zhenjiang1-7 | 41 | tetravalent vaccine | N | A B Mi P |
| Zhenjiang1-8 | 42 | tetravalent vaccine | N | A |
| Zhenjiang1-9 | 42 | tetravalent vaccine | N | A N T P |
| Zhenjiang1-10 | 43 | tetravalent vaccine | N | A N Mi |
| Zhenjiang1-11 | 44 | tetravalent vaccine | Y | A N |
| Zhenjiang1-12 | 44 | tetravalent vaccine | Y | A T Mi |
| Lianyungang1-1 | 53 | tetravalent vaccine | N | A B N T Mi P |
| Lianyungang1-2 | 49 | tetravalent vaccine | N | A B N T Mi P |
| Lianyungang1-3 | 55 | tetravalent vaccine | N | nd |
| Lianyungang1-4 | 49 | tetravalent vaccine | Y | A B N T P |
| Lianyungang1-5 | 38 | tetravalent vaccine | N | A N Mi P |
| Lianyungang1-6 | 46 | tetravalent vaccine | Y | A T N Mi P |
| Lianyungang1-7 | 51 | tetravalent vaccine | N | A B N P |
| Lianyungang1-8 | 37 | tetravalent vaccine | N | A T |
| Lianyungang1-9 | 39 | tetravalent vaccine | N | A N T |
| Shaoxing1-1 | 27 | tetravalent vaccine | N | A N Mi P |
| Shaoxing1-2 | 26 | tetravalent vaccine | N | A N T |
| Shaoxing1-3 | 22 | tetravalent vaccine | N | A |
| Shaoxing1-4 | 25 | tetravalent vaccine | N | A N T Ma Mi P |
| Shaoxing1-5 | 53 | tetravalent vaccine | N | nd |
| Shaoxing1-6 | 47 | tetravalent vaccine | N | A N P |
| Shaoxing1-7 | 58 | tetravalent vaccine | N | A N T P |

to be continued

| **District** | **Age (days)** | **Vaccine** | **Clinic** | **Coccidia species** |
| --- | --- | --- | --- | --- |
| Shaoxing1-8 | 41 | tetravalent vaccine | N | A N T Mi P |
| Shaoxing1-9 | 59 | tetravalent vaccine | N | A N T Mi P |
| Shaoxing1-10 | 32 | tetravalent vaccine | N | nd |
| Shaoxing1-11 | 41 | tetravalent vaccine | N | A N T Mi |
| Shaoxing1-12 | 31 | tetravalent vaccine | N | A N T Mi |
| Shaoxing1-13 | 41 | tetravalent vaccine | N | A N T Mi P |
| Shaoxing1-14 | 56 | tetravalent vaccine | N | A N T Mi P |
| Shaoxing1-15 | 34 | tetravalent vaccine | N | A N T Mi P |
| Huzhou1-1 | 36 | trivalent vaccine | N | A N T Mi |
| Huzhou1-2 | 32 | tetravalent vaccine | N | A T Mi P |
| Huzhou1-3 | 58 | tetravalent vaccine | Y | N P |
| Huzhou1-4 | 70 | tetravalent vaccine | N | N |
| Huzhou1-5 | 27 | trivalent vaccine | N | A B N T Mi P |
| Huzhou1-6 | 34 | tetravalent vaccine | Y | A N Mi P |
| Huzhou1-7 | 52 | trivalent vaccine | N | A T P |
| Huzhou1-8 | 54 | trivalent vaccine | Y | N Mi |
| Huzhou1-9 | 29 | trivalent vaccine | N | A B T Mi P |
| Huzhou1-10 | 27 | trivalent vaccine | Y | A T Mi P |
| Huzhou1-11 | 73 | trivalent vaccine | N | N |
| Huzhou1-12 | 57 | tetravalent vaccine | N | N |
| Wuhu1-1 | 40 | trivalent vaccine | Y | A Mi P |
| Wuhu1-2 | 36 | trivalent vaccine | N | A |
| Wuhu1-3 | 40 | trivalent vaccine | Y | A B Mi |
| Wuhu1-4 | 45 | trivalent vaccine | N | nd |
| Wuhu1-5 | 44 | trivalent vaccine | N | A B N Mi |
| Wuhu1-6 | 28 | trivalent vaccine | N | A T Mi |
| Wuhu1-7 | 28 | trivalent vaccine | Y | A B T Mi P |
| Wuhu1-8 | 31 | trivalent vaccine | N | N Mi P |
| Wuhu1-9 | 26 | trivalent vaccine | N | nd |
| Wuhu1-10 | 29 | trivalent vaccine | N | A N Mi P |
| Wuhu1-11 | 66 | trivalent vaccine | N | A N Mi P |
| Wuhu1-12 | 28 | trivalent vaccine | N | A |
| Wuhu1-13 | 29 | trivalent vaccine | Y | A N Mi P |
| Wuhu1-14 | 56 | trivalent vaccine | N | A Mi |
| Wuhu1-15 | 57 | trivalent vaccine | Y | A Mi |
| Chuzhou1-1 | 54 | tetravalent vaccine | N | A N |
| Chuzhou1-2 | 52 | tetravalent vaccine | N | A N T Mi |
| Chuzhou1-3 | 22 | tetravalent vaccine | N | A B N T Mi |
| Chuzhou1-4 | 24 | tetravalent vaccine | Y | A N T |
| Chuzhou1-5 | 27 | tetravalent vaccine | N | A B N Mi |
| Chuzhou1-6 | 39 | tetravalent vaccine | N | A N Mi |
| Chuzhou1-7 | 38 | tetravalent vaccine | Y | A N Mi P |

to be continued

| **District** | **Age (days)** | **Vaccine** | **Clinic** | **Coccidia species** |
| --- | --- | --- | --- | --- |
| Chuzhou1-8 | 39 | tetravalent vaccine | N | A N Mi P |
| Chuzhou1-9 | 58 | tetravalent vaccine | N | A N T Mi |
| Chuzhou1-10 | 70 | tetravalent vaccine | N | A B N T Mi P |
| Chuzhou1-11 | 73 | tetravalent vaccine | N | A N T Ma Mi |
| Jian1-1 | 47 | tetravalent vaccine | N | A B N T Mi P |
| Jian1-2 | 27 | tetravalent vaccine | Y | A N T Mi P |
| Jian1-3 | 22 | tetravalent vaccine | N | A N |
| Jian1-4 | 49 | tetravalent vaccine | N | A B T Mi P |
| Jian1-5 | 27 | tetravalent vaccine | Y | A T Mi |
| Jian1-6 | 50 | tetravalent vaccine | N | A B Mi P |
| Jian1-7 | 51 | tetravalent vaccine | Y | N T |
| Jian1-8 | 40 | tetravalent vaccine | N | A B T Mi |
| Jian1-9 | 25 | tetravalent vaccine | Y | A N T |
| Jian1-10 | 46 | tetravalent vaccine | Y | A B T Mi P |
| Jian1-11 | 43 | tetravalent vaccine | N | A N Mi |
| Jian1-12 | 43 | tetravalent vaccine | Y | A T Mi |
| Jian1-13 | 38 | tetravalent vaccine | N | A B Ma Mi |
| Jian1-14 | 33 | tetravalent vaccine | N | A Mi P |
| Jian1-15 | 32 | tetravalent vaccine | N | A N T |
| Jian1-16 | 30 | tetravalent vaccine | N | A N Mi |
| Taian1-1 | 38 | tetravalent vaccine | N | A B Mi P |
| Taian1-2 | 54 | tetravalent vaccine | N | A N T Mi P |
| Taian1-3 | 41 | trivalent vaccine | N | A N T Mi P |
| Taian1-4 | 42 | tetravalent vaccine | Y | A B N T Mi P |
| Taian1-5 | 36 | tetravalent vaccine | N | Mi |
| Taian1-6 | 64 | tetravalent vaccine | Y | A N T |
| Taian1-7 | 63 | tetravalent vaccine | Y | B N Mi P |
| Taian1-8 | 41 | tetravalent vaccine | N | A N T |
| Taian1-9 | 22 | tetravalent vaccine | N | A N T Mi |
| Taian1-10 | 29 | tetravalent vaccine | Y | A B N T Mi P |
| Taian1-11 | 40 | trivalent vaccine | N | A N T |
| Taian1-12 | 39 | tetravalent vaccine | N | A T Mi P |
| Taian1-13 | 23 | tetravalent vaccine | N | A Mi P |
| Nanping1-1 | 27 | tetravalent vaccine | N | nd |
| Nanping1-2 | 48 | tetravalent vaccine | Y | N Mi P |
| Nanping1-3 | 35 | tetravalent vaccine | N | A B N T |
| Nanping1-4 | 28 | tetravalent vaccine | N | A T Mi P |
| Nanping1-5 | 71 | tetravalent vaccine | N | nd |
| Nanping1-6 | 40 | tetravalent vaccine | N | B N T Mi P |
| Nanping1-7 | 58 | tetravalent vaccine | N | nd |
| Nanping1-8 | 58 | tetravalent vaccine | Y | A B N T Mi P |
| Nanping1-9 | 45 | tetravalent vaccine | Y | A N T Mi P |

to be continued

| **District** | **Age (days)** | **Vaccine** | **Clinic** | **Coccidia species** |
| --- | --- | --- | --- | --- |
| Nanping1-10 | 38 | tetravalent vaccine | N | nd |
| Nanping1-11 | 29 | tetravalent vaccine | N | nd |
| Nanping1-12 | 51 | tetravalent vaccine | N | A N T Mi P |
| Nanping1-13 | 40 | tetravalent vaccine | N | N T Mi |
| Nanping1-14 | 39 | tetravalent vaccine | N | nd |
| Nanping1-15 | 32 | tetravalent vaccine | N | Mi |
| Nanping1-16 | 32 | tetravalent vaccine | N | A T Mi |
| Putian1-1 | 24 | tetravalent vaccine | N | A N T Mi |
| Putian1-2 | 41 | tetravalent vaccine | N | A T Mi |
| Putian1-3 | 50 | tetravalent vaccine | N | nd |
| Putian1-4 | 50 | tetravalent vaccine | N | A N Mi |
| Putian1-5 | 23 | tetravalent vaccine | N | A N T Ma |
| Putian1-6 | 42 | tetravalent vaccine | Y | A T |
| Putian1-7 | 46 | tetravalent vaccine | N | nd |
| Putian1-8 | 33 | tetravalent vaccine | N | A B Mi P |
| Putian1-9 | 24 | tetravalent vaccine | N | N |
| Putian1-10 | 45 | tetravalent vaccine | Y | Mi P |
| Putian1-11 | 35 | tetravalent vaccine | N | Mi P |
| Putian1-12 | 42 | tetravalent vaccine | N | Mi P |

**Table S2.** The results of the identification of *Eimeria* species in central China.

| **District** | **Age (days)** | **Vaccine** | **Clinic** | **Coccidia species** |
| --- | --- | --- | --- | --- |
| Sanmenxia1-1 | 58 | tetravalent vaccine | N | N T Mi P |
| Sanmenxia1-2 | 25 | tetravalent vaccine | N | N T Mi |
| Sanmenxia1-3 | 45 | tetravalent vaccine | N | T |
| Sanmenxia1-4 | 50 | tetravalent vaccine | N | T |
| Sanmenxia1-5 | 32 | tetravalent vaccine | N | N T Mi |
| Sanmenxia1-6 | 36 | tetravalent vaccine | N | A N T P |
| Sanmenxia1-7 | 40 | tetravalent vaccine | N | Mi P |
| Sanmenxia1-8 | 33 | tetravalent vaccine | N | B T Mi |
| Sanmenxia1-9 | 53 | tetravalent vaccine | N | T Mi P |
| Sanmenxia1-10 | 28 | tetravalent vaccine | N | T Mi P |
| Sanmenxia1-11 | 48 | tetravalent vaccine | N | T Mi |
| Sanmenxia1-12 | 55 | tetravalent vaccine | Y | P |
| Sanmenxia1-13 | 21 | tetravalent vaccine | N | N |
| Xianning1-1 | 23 | tetravalent vaccine | N | A N T Mi P |
| Xianning1-2 | 48 | tetravalent vaccine | N | A N T Mi P |
| Xianning1-3 | 54 | tetravalent vaccine | Y | A B N T Mi P |
| Xianning1-4 | 43 | tetravalent vaccine | N | A B N T Mi P |
| Xianning1-5 | 42 | tetravalent vaccine | Y | A B T |
| Xianning1-6 | 20 | tetravalent vaccine | N | A N T Mi |
| Xianning1-7 | 42 | tetravalent vaccine | Y | A B T Mi P |
| Xianning1-8 | 33 | tetravalent vaccine | N | A N T Mi P |
| Xianning1-9 | 61 | tetravalent vaccine | N | A B N T Mi P |
| Xianning1-10 | 35 | tetravalent vaccine | N | A N T Mi |
| Xianning1-11 | 39 | tetravalent vaccine | N | A T Mi |
| Xiaogan1-1 | 45 | tetravalent vaccine | N | nd |
| Xiaogan1-2 | 42 | tetravalent vaccine | Y | A B N T Mi |
| Xiaogan1-3 | 42 | tetravalent vaccine | N | A B N T Mi |
| Xiaogan1-4 | 32 | tetravalent vaccine | Y | T |
| Xiaogan1-5 | 35 | tetravalent vaccine | Y | A N Mi |
| Xiaogan1-6 | 50 | tetravalent vaccine | N | A T Mi |
| Xiaogan1-7 | 36 | tetravalent vaccine | N | A B N T Mi |
| Xiaogan1-8 | 49 | tetravalent vaccine | N | A N T |
| Xiaogan1-9 | 63 | tetravalent vaccine | N | N P |
| Xiaogan1-10 | 28 | tetravalent vaccine | N | A T Mi |
| Xiaogan1-11 | 22 | tetravalent vaccine | N | A N T |
| Xiaogan1-12 | 23 | tetravalent vaccine | N | A N |
| Xiaogan1-13 | 58 | tetravalent vaccine | N | A N P |
| Changsha1-1 | 45 | tetravalent vaccine | N | A P |
| Changsha1-2 | 46 | tetravalent vaccine | N | A B N T Mi P |
| Changsha1-3 | 47 | tetravalent vaccine | N | nd |

to be continued

| **District** | **Age (days)** | **Vaccine** | **Clinic** | **Coccidia species** |
| --- | --- | --- | --- | --- |
| Changsha1-4 | 37 | tetravalent vaccine | N | T |
| Changsha1-5 | 22 | tetravalent vaccine | N | A N T |
| Changsha1-6 | 35 | tetravalent vaccine | N | nd |
| Changsha1-7 | 28 | tetravalent vaccine | N | T |
| Changsha1-8 | 60 | tetravalent vaccine | N | A T P |
| Changsha1-9 | 29 | tetravalent vaccine | N | A T Mi |
| Changsha1-10 | 47 | tetravalent vaccine | N | A T |
| Changsha1-11 | 47 | tetravalent vaccine | N | A B T Mi P |
| Changsha1-12 | 32 | tetravalent vaccine | N | Mi P |
| Hengyang1-1 | 21 | tetravalent vaccine | N | A N T Mi P |
| Hengyang1-2 | 25 | tetravalent vaccine | N | N T |
| Hengyang1-3 | 41 | tetravalent vaccine | N | A T Mi P |
| Hengyang1-4 | 28 | tetravalent vaccine | N | A N |
| Hengyang1-5 | 31 | tetravalent vaccine | N | N Mi P |
| Hengyang1-6 | 47 | tetravalent vaccine | N | A N T Mi P |
| Hengyang1-7 | 40 | tetravalent vaccine | N | A |
| Hengyang1-8 | 34 | tetravalent vaccine | N | A N P |
| Hengyang1-9 | 42 | tetravalent vaccine | N | A N T Mi P |
| Hengyang1-10 | 32 | tetravalent vaccine | N | A N T |
| Hengyang1-11 | 44 | tetravalent vaccine | N | P |
| Hengyang1-12 | 48 | tetravalent vaccine | N | A N Mi P |
| Linxiang1-1 | 30 | tetravalent vaccine | N | A N T Mi |
| Linxiang1-2 | 40 | tetravalent vaccine | N | A B N T Mi |
| Linxiang1-3 | 32 | tetravalent vaccine | N | A T |
| Linxiang1-4 | 44 | tetravalent vaccine | N | A N Mi P |
| Linxiang1-5 | 50 | tetravalent vaccine | Y | A B N T Mi P |
| Linxiang1-6 | 38 | tetravalent vaccine | N | A T |
| Linxiang1-7 | 38 | tetravalent vaccine | N | A N T |
| Linxiang1-8 | 43 | tetravalent vaccine | Y | A Ma Mi |
| Linxiang1-9 | 28 | tetravalent vaccine | N | A B T Mi P |
| Linxiang1-10 | 24 | tetravalent vaccine | N | A N T Ma |
| Linxiang1-11 | 26 | tetravalent vaccine | Y | A N T Ma |
| Linxiang1-12 | 54 | tetravalent vaccine | Y | N T Mi P |
| Linxiang1-13 | 27 | tetravalent vaccine | Y | A B T Mi P |
| Linxiang1-14 | 36 | tetravalent vaccine | N | A T |
| Linxiang1-15 | 41 | tetravalent vaccine | N | A T |
| Linxiang1-16 | 30 | tetravalent vaccine | N | A B T Ma Mi P |
| Linxiang1-17 | 47 | tetravalent vaccine | N | A N T Mi P |
| Linxiang1-18 | 33 | tetravalent vaccine | Y | A B T Ma Mi P |

to be continued

**Table S3.** The results of the identification of *Eimeria* species in south China.

| **District** | **Age (days)** | **Vaccine** | **Clinic** | **Coccidia species** |
| --- | --- | --- | --- | --- |
| Meizhou1-1 | 46 | tetravalent vaccine | N | A N |
| Meizhou1-2 | 46 | tetravalent vaccine | N | A B N Mi |
| Meizhou1-3 | 54 | tetravalent vaccine | N | nd |
| Meizhou1-4 | 37 | tetravalent vaccine | N | N |
| Meizhou1-5 | 46 | tetravalent vaccine | N | nd |
| Meizhou1-6 | 43 | tetravalent vaccine | N | A B N |
| Meizhou1-7 | 51 | tetravalent vaccine | N | A B Mi |
| Meizhou1-8 | 25 | tetravalent vaccine | N | nd |
| Meizhou1-9 | 21 | tetravalent vaccine | N | N |
| Meizhou1-10 | 24 | tetravalent vaccine | N | A Mi |
| Meizhou1-11 | 34 | tetravalent vaccine | N | nd |
| Meizhou1-12 | 31 | tetravalent vaccine | N | nd |
| Meizhou1-13 | 26 | tetravalent vaccine | N | N |
| Heyuan1-1 | 59 | tetravalent vaccine | Y | A N Mi |
| Heyuan1-2 | 56 | tetravalent vaccine | N | nd |
| Heyuan1-3 | 61 | tetravalent vaccine | Y | B N |
| Heyuan1-4 | 55 | tetravalent vaccine | Y | N |
| Heyuan1-5 | 47 | tetravalent vaccine | Y | A N T |
| Heyuan1-6 | 49 | tetravalent vaccine | Y | A B N Mi P |
| Heyuan1-7 | 36 | tetravalent vaccine | N | A B T Mi P |
| Heyuan1-8 | 37 | tetravalent vaccine | N | nd |
| Heyuan1-9 | 34 | tetravalent vaccine | N | A B Mi P |
| Heyuan1-10 | 29 | tetravalent vaccine | Y | A T Mi P |
| Heyuan1-11 | 27 | tetravalent vaccine | Y | A N T |
| Heyuan1-12 | 27 | trivalent vaccine | N | A T Mi P |
| Heyuan1-13 | 25 | trivalent vaccine | N | A T P |
| Heyuan1-14 | 27 | trivalent vaccine | Y | N Ma Mi |
| Heyuan1-15 | 29 | trivalent vaccine | N | A B T Mi P |
| Heyuan1-16 | 31 | trivalent vaccine | Y | A T Mi P |
| Heyuan1-17 | 60 | trivalent vaccine | Y | N Mi P |
| Heyuan1-18 | 60 | trivalent vaccine | Y | N |
| Heyuan1-13 | 32 | tetravalent vaccine | N | A B T P |
| Heyuan1-14 | 43 | tetravalent vaccine | N | A N Mi P |
| Heyuan2-1 | 21 | tetravalent vaccine | N | A N T P |
| Heyuan2-2 | 35 | tetravalent vaccine | N | A N Mi P |
| Heyuan2-3 | 20 | tetravalent vaccine | N | A N T P |
| Heyuan2-4 | 27 | tetravalent vaccine | N | A B N T Mi P |
| Heyuan2-5 | 41 | tetravalent vaccine | N | A B N T Mi P |
| Heyuan2-6 | 50 | tetravalent vaccine | N | A |
| Heyuan2-7 | 20 | tetravalent vaccine | N | A T |
| Heyuan2-8 | 46 | tetravalent vaccine | Y | A N T Mi |

to be continued

| **District** | **Age (days)** | **Vaccine** | **Clinic** | **Coccidia species** |
| --- | --- | --- | --- | --- |
| Heyuan2-9 | 36 | tetravalent vaccine | N | A N T P |
| Heyuan2-10 | 43 | tetravalent vaccine | N | A B N T Mi P |
| Heyuan2-11 | 43 | tetravalent vaccine | N | N Mi |
| Xinxing1-1 | 25 | trivalent vaccine | N | A Ma Mi P |
| Xinxing1-2 | 75 | tetravalent vaccine | N | nd |
| Xinxing1-3 | 26 | tetravalent vaccine | N | A T Mi |
| Xinxing1-4 | 50 | tetravalent vaccine | N | A B N T |
| Xinxing1-5 | 46 | trivalent vaccine | N | A B N T Mi P |
| Xinxing1-6 | 27 | trivalent vaccine | N | A T Mi P |
| Xinxing1-7 | 38 | trivalent vaccine | N | A B N T Mi |
| Xinxing1-8 | 73 | tetravalent vaccine | N | N T |
| Xinxing1-9 | 68 | tetravalent vaccine | N | A B N T |
| Xinxing1-10 | 46 | tetravalent vaccine | N | A N T Mi P |
| Xinxing1-11 | 40 | tetravalent vaccine | N | A B N T Mi P |
| Xinxing1-12 | 36 | tetravalent vaccine | N | A N T Mi P |
| Xinxing2-1 | 26 | trivalent vaccine | Y | A Ma Mi P |
| Xinxing2-2 | 26 | trivalent vaccine | N | A |
| Xinxing2-3 | 43 | trivalent vaccine | Y | A B Mi |
| Xinxing2-4 | 45 | trivalent vaccine | N | A B |
| Xinxing2-5 | 45 | trivalent vaccine | N | A B N Mi |
| Xinxing2-6 | 25 | trivalent vaccine | N | A T Mi |
| Xinxing2-7 | 25 | trivalent vaccine | Y | A T Ma Mi P |
| Xinxing2-8 | 31 | trivalent vaccine | Y | A T Mi P |
| Xinxing2-9 | 26 | trivalent vaccine | N | nd |
| Xinxing2-10 | 60 | trivalent vaccine | N | nd |
| Xinxing2-11 | 66 | trivalent vaccine | N | nd |
| Xinxing2-12 | 60 | trivalent vaccine | N | A B |
| Yangjiang1-1 | 34 | tetravalent vaccine | N | A N Mi |
| Yangjiang1-2 | 34 | tetravalent vaccine | N | A T Mi P |
| Yangjiang1-3 | 22 | tetravalent vaccine | N | T Mi |
| Yangjiang1-4 | 43 | tetravalent vaccine | N | A Mi |
| Yangjiang1-5 | 48 | tetravalent vaccine | N | nd |
| Yangjiang1-6 | 24 | tetravalent vaccine | N | A Mi P |
| Yangjiang1-7 | 80 | tetravalent vaccine | N | nd |
| Yangjiang1-8 | 19 | tetravalent vaccine | N | nd |
| Yangjiang1-9 | 44 | tetravalent vaccine | N | nd |
| Yangjiang1-10 | 45 | tetravalent vaccine | N | nd |
| Yangjiang1-11 | 34 | tetravalent vaccine | N | A Mi P |
| Yangjiang1-12 | 25 | tetravalent vaccine | N | nd |
| Yangjiang1-13 | 34 | tetravalent vaccine | N | A T Ma Mi P |
| Yangjiang1-14 | 22 | tetravalent vaccine | N | nd |
| Yangjiang1-15 | 34 | tetravalent vaccine | N | nd |

to be continued

| **District** | **Age (days)** | **Vaccine** | **Clinic** | **Coccidia species** |
| --- | --- | --- | --- | --- |
| Yunfu1-1 | 26 | tetravalent vaccine | N | nd |
| Yunfu1-2 | 36 | trivalent vaccine | N | nd |
| Yunfu1-3 | 72 | tetravalent vaccine | N | nd |
| Yunfu1-4 | 40 | tetravalent vaccine | N | nd |
| Yunfu1-5 | 41 | tetravalent vaccine | Y | A N T Mi P |
| Yunfu1-6 | 76 | tetravalent vaccine | N | nd |
| Yunfu1-7 | 30 | tetravalent vaccine | N | A Mi |
| Yunfu1-8 | 20 | tetravalent vaccine | Y | N |
| Yunfu1-9 | 37 | tetravalent vaccine | N | N |
| Yunfu1-10 | 60 | tetravalent vaccine | N | A |
| Yunfu1-11 | 44 | tetravalent vaccine | N | N |
| Yunfu1-12 | 45 | tetravalent vaccine | N | N |
| Yunfu2-1 | 27 | tetravalent vaccine | N | A N T Mi P |
| Yunfu2-2 | 40 | tetravalent vaccine | N | A B N T Mi P |
| Yunfu2-3 | 50 | tetravalent vaccine | N | N |
| Yunfu2-4 | 49 | tetravalent vaccine | Y | A N T |
| Yunfu2-5 | 55 | tetravalent vaccine | N | N T Ma Mi |
| Yunfu2-6 | 53 | tetravalent vaccine | N | N T |
| Yunfu2-7 | 72 | tetravalent vaccine | N | nd |
| Yunfu2-8 | 32 | tetravalent vaccine | Y | N T Mi |
| Yunfu2-9 | 26 | tetravalent vaccine | N | A N T Mi |
| Yunfu2-10 | 49 | tetravalent vaccine | N | nd |
| Yunfu2-11 | 46 | tetravalent vaccine | Y | N T Mi |
| Yunfu2-12 | 96 | tetravalent vaccine | N | nd |
| Yunfu2-13 | 35 | tetravalent vaccine | N | A B T Mi P |
| Yunfu2-14 | 48 | tetravalent vaccine | N | A Mi P |
| Yunfu2-15 | 51 | tetravalent vaccine | N | A N T Mi P |
| Yunfu2-16 | 34 | tetravalent vaccine | N | N |
| Yunfu2-17 | 30 | tetravalent vaccine | N | B T Mi P |
| Yunfu2-18 | 48 | trivalent vaccine | N | A T Mi |
| Yunfu3-1 | 27 | trivalent vaccine | Y | T Mi |
| Yunfu3-2 | 54 | trivalent vaccine | N | A N T Mi P |
| Yunfu3-3 | 39 | trivalent vaccine | Y | A Mi P |
| Yunfu3-4 | 23 | trivalent vaccine | Y | N T |
| Yunfu3-5 | 55 | trivalent vaccine | N | N |
| Yunfu3-6 | 46 | trivalent vaccine | Y | A N Mi |
| Yunfu3-7 | 40 | trivalent vaccine | Y | T Mi |
| Yunfu3-8 | 36 | trivalent vaccine | Y | A N T Mi P |
| Yunfu3-9 | 29 | trivalent vaccine | N | A Mi P |
| Yunfu3-10 | 45 | trivalent vaccine | Y | N T |
| Yunfu3-11 | 49 | trivalent vaccine | N | A N T Mi |
| Foshan1-1 | 26 | tetravalent vaccine | N | A |

to be continued

| **District** | **Age (days)** | **Vaccine** | **Clinic** | **Coccidia species** |
| --- | --- | --- | --- | --- |
| Foshan1-2 | 36 | tetravalent vaccine | N | A N T Mi P |
| Foshan1-3 | 25 | tetravalent vaccine | Y | A N P |
| Foshan1-4 | 49 | tetravalent vaccine | N | A T Mi P |
| Foshan1-5 | 42 | tetravalent vaccine | N | nd |
| Foshan1-6 | 19 | tetravalent vaccine | Y | A N Mi |
| Foshan1-7 | 39 | tetravalent vaccine | N | A N T Mi P |
| Foshan1-8 | 59 | tetravalent vaccine | N | A N |
| Foshan1-9 | 43 | tetravalent vaccine | N | T |
| Foshan1-10 | 49 | tetravalent vaccine | N | A N T P |
| Foshan1-11 | 61 | tetravalent vaccine | N | A N T Mi P |
| Foshan1-12 | 48 | tetravalent vaccine | N | nd |
| Foshan2-1 | 41 | tetravalent vaccine | Y | A T Mi P |
| Foshan2-2 | 20 | tetravalent vaccine | Y | A N T P |
| Foshan2-3 | 65 | tetravalent vaccine | N | nd |
| Foshan2-4 | 55 | tetravalent vaccine | Y | A B N Mi |
| Foshan2-5 | 39 | tetravalent vaccine | Y | A B N T Ma Mi P |
| Foshan2-6 | 53 | tetravalent vaccine | N | A N Mi |
| Foshan2-7 | 20 | tetravalent vaccine | N | A N P |
| Foshan2-8 | 34 | tetravalent vaccine | Y | A N Mi P |
| Foshan2-9 | 43 | tetravalent vaccine | Y | A T P |
| Foshan2-10 | 77 | tetravalent vaccine | Y | A N T Mi P |
| Foshan2-11 | 46 | tetravalent vaccine | Y | A B T Mi |
| Foshan2-12 | 30 | tetravalent vaccine | Y | A N T Ma |
| Zhaoqing1-1 | 13 | tetravalent vaccine | N | N T |
| Zhaoqing1-2 | 21 | tetravalent vaccine | N | A T |
| Zhaoqing1-3 | 63 | tetravalent vaccine | Y | A |
| Zhaoqing1-4 | 48 | tetravalent vaccine | N | nd |
| Zhaoqing1-5 | 60 | tetravalent vaccine | Y | A B |
| Zhaoqing1-6 | 29 | tetravalent vaccine | N | B |
| Zhaoqing1-7 | 58 | tetravalent vaccine | N | nd |
| Zhaoqing1-8 | 35 | tetravalent vaccine | N | B N T |
| Zhaoqing1-9 | 18 | tetravalent vaccine | N | B T |
| Zhaoqing1-10 | 21 | tetravalent vaccine | N | A N T P |
| Zhaoqing1-11 | 21 | tetravalent vaccine | N | A N T Ma Mi |
| Zhaoqing1-12 | 47 | tetravalent vaccine | N | nd |
| Jiangmen1-1 | 49 | tetravalent vaccine | Y | A Mi |
| Jiangmen1-2 | 56 | tetravalent vaccine | Y | A N T Mi P |
| Jiangmen1-3 | 33 | tetravalent vaccine | Y | A N T Mi P |
| Jiangmen1-4 | 65 | tetravalent vaccine | Y | A T |
| Jiangmen1-5 | 22 | tetravalent vaccine | N | A B N T P |
| Jiangmen1-6 | 32 | tetravalent vaccine | Y | A B N T Mi P |

to be continued

| **District** | **Age (days)** | **Vaccine** | **Clinic** | **Coccidia species** |
| --- | --- | --- | --- | --- |
| Jiangmen1-7 | 27 | tetravalent vaccine | Y | A B Mi P |
| Jiangmen1-8 | 31 | tetravalent vaccine | Y | A N |
| Jiangmen1-9 | 29 | tetravalent vaccine | Y | N T Mi P |
| Jiangmen1-10 | 40 | tetravalent vaccine | Y | N T P |
| Jiangmen1-11 | 27 | tetravalent vaccine | Y | A B N T |
| Jiangmen1-12 | 31 | tetravalent vaccine | Y | N T Mi P |
| Nanning1-1 | 56 | tetravalent vaccine | Y | P |
| Nanning1-2 | 64 | tetravalent vaccine | Y | N P |
| Nanning1-3 | 44 | tetravalent vaccine | Y | T |
| Nanning1-4 | 54 | tetravalent vaccine | N | N |
| Nanning1-5 | 58 | tetravalent vaccine | Y | A N Mi |
| Nanning1-6 | 70 | tetravalent vaccine | N | B N |
| Nanning1-7 | 64 | tetravalent vaccine | Y | N T P |
| Nanning1-8 | 58 | tetravalent vaccine | Y | A B P |
| Nanning1-9 | 48 | tetravalent vaccine | Y | A P |
| Nanning1-10 | 52 | tetravalent vaccine | Y | A B P |
| Nanning2-1 | 41 | trivalent vaccine | Y | A N |
| Nanning2-2 | 36 | trivalent vaccine | N | B N T Mi |
| Nanning2-3 | 35 | trivalent vaccine | N | A B Mi P |
| Nanning2-4 | 42 | trivalent vaccine | Y | A B N T Mi |
| Nanning2-5 | 41 | trivalent vaccine | N | A B N T Mi |
| Nanning2-6 | 40 | trivalent vaccine | Y | A N Mi |
| Nanning2-7 | 27 | trivalent vaccine | N | A B N Mi |
| Nanning2-8 | 24 | trivalent vaccine | N | A N T Mi |
| Nanning2-9 | 39 | trivalent vaccine | N | A T Mi P |
| Nanning2-10 | 21 | trivalent vaccine | N | A Mi P |
| Nanning2-11 | 59 | trivalent vaccine | Y | A N Mi |
| Nanning2-12 | 59 | trivalent vaccine | Y | A N Mi |
| Nanning2-13 | 25 | trivalent vaccine | Y | A Mi P |
| Nanning2-14 | 46 | trivalent vaccine | N | A Mi |
| Nanning2-15 | 47 | trivalent vaccine | Y | N Mi |
| Hezhou1-1 | 42 | tetravalent vaccine | N | A |
| Hezhou1-2 | 56 | tetravalent vaccine | N | A N T |
| Hezhou1-3 | 53 | tetravalent vaccine | N | A N T P |
| Hezhou1-4 | 51 | tetravalent vaccine | N | A N T |
| Hezhou1-5 | 39 | tetravalent vaccine | N | A B N T Mi P |
| Hezhou1-6 | 39 | tetravalent vaccine | N | B T |
| Hezhou1-7 | 52 | tetravalent vaccine | N | N P |
| Hezhou1-8 | 22 | tetravalent vaccine | N | N |
| Hezhou1-9 | 33 | tetravalent vaccine | N | A B T Mi P |
| Hezhou1-10 | 26 | tetravalent vaccine | N | N T |

to be continued

| **District** | **Age (days)** | **Vaccine** | **Clinic** | **Coccidia species** |
| --- | --- | --- | --- | --- |
| Hezhou1-11 | 51 | tetravalent vaccine | N | N T P |
| Hezhou1-12 | 22 | tetravalent vaccine | N | A N T Ma |
| Yulin1-1 | 44 | tetravalent vaccine | N | A N T P |
| Yulin1-2 | 34 | tetravalent vaccine | N | N T |
| Yulin1-3 | 43 | tetravalent vaccine | N | nd |
| Yulin1-4 | 23 | tetravalent vaccine | Y | N T P |
| Yulin1-5 | 34 | tetravalent vaccine | Y | B N P |
| Yulin1-6 | 27 | tetravalent vaccine | N | B N T |
| Yulin1-7 | 36 | tetravalent vaccine | Y | N P |
| Yulin1-8 | 22 | tetravalent vaccine | N | nd |
| Yulin1-9 | 35 | tetravalent vaccine | Y | N T |
| Yulin1-10 | 23 | tetravalent vaccine | Y | B N T Ma P |
| Yulin1-11 | 34 | tetravalent vaccine | N | B N T P |
| Yulin1-12 | 46 | tetravalent vaccine | N | B N T P |
| Yulin1-13 | 41 | tetravalent vaccine | Y | B P |
| Yulin2-1 | 44 | tetravalent vaccine | N | A B N T |
| Yulin2-2 | 46 | tetravalent vaccine | Y | A N T P |
| Yulin2-3 | 44 | tetravalent vaccine | N | B N T |
| Yulin2-4 | 29 | tetravalent vaccine | N | nd |
| Yulin2-5 | 25 | tetravalent vaccine | N | A B T Mi P |
| Yulin2-6 | 34 | tetravalent vaccine | N | A B T Mi P |
| Yulin2-7 | 44 | tetravalent vaccine | N | A B N T Mi P |
| Yulin2-8 | 45 | tetravalent vaccine | N | A N T Mi P |
| Yulin2-9 | 52 | tetravalent vaccine | Y | A N T Mi P |
| Yulin2-10 | 42 | tetravalent vaccine | N | A N T Mi |
| Yulin2-11 | 45 | tetravalent vaccine | N | A Mi |
| Yulin2-12 | 46 | tetravalent vaccine | N | A T Mi |
| Yulin2-13 | 45 | tetravalent vaccine | Y | A N Mi |

**Table S4.** The results of the identification of *Eimeria* species in southwest China.

| **District** | **Age (days)** | **Vaccine** | **Clinic** | **Coccidia species** |
| --- | --- | --- | --- | --- |
| Bishan1-1 | 37 | tetravalent vaccine | N | A |
| Bishan1-2 | 32 | tetravalent vaccine | N | A Mi P |
| Bishan1-3 | 41 | tetravalent vaccine | N | Mi |
| Bishan1-4 | 32 | tetravalent vaccine | N | A N T Mi P |
| Bishan1-5 | 21 | tetravalent vaccine | N | nd |
| Bishan1-6 | 23 | tetravalent vaccine | N | A B N T |
| Bishan1-7 | 20 | tetravalent vaccine | N | A T Mi P |
| Bishan1-8 | 52 | tetravalent vaccine | Y | A |
| Bishan1-9 | 68 | tetravalent vaccine | N | nd |
| Bishan1-10 | 31 | tetravalent vaccine | N | A B N Mi P |
| Bishan1-11 | 57 | tetravalent vaccine | N | A Mi |
| Bishan1-12 | 61 | tetravalent vaccine | Y | A B |
| Dazhou1-1 | 50 | tetravalent vaccine | Y | A P |
| Dazhou1-2 | 46 | tetravalent vaccine | N | A |
| Dazhou1-3 | 30 | tetravalent vaccine | Y | A T Mi P |
| Dazhou1-4 | 24 | tetravalent vaccine | N | A B N T P |
| Dazhou1-5 | 57 | tetravalent vaccine | N | N Mi P |
| Dazhou1-6 | 20 | tetravalent vaccine | N | A N T Mi |
| Dazhou1-7 | 46 | tetravalent vaccine | Y | A T |
| Dazhou1-8 | 54 | tetravalent vaccine | N | A N |
| Dazhou1-9 | 55 | tetravalent vaccine | Y | T |
| Dazhou1-10 | 46 | tetravalent vaccine | N | B Mi P |
| Dazhou1-11 | 29 | tetravalent vaccine | N | A B T Mi P |
| Dazhou1-12 | 67 | tetravalent vaccine | Y | A B Mi P |
| Dazhou1-13 | 64 | tetravalent vaccine | Y | A |
| Dazhou1-14 | 32 | tetravalent vaccine | N | A T P |
| Dazhou1-15 | 41 | tetravalent vaccine | N | A |
| Meizhou1-1 | 46 | tetravalent vaccine | N | nd |
| Meizhou1-2 | 33 | tetravalent vaccine | N | A Mi |
| Meizhou1-3 | 41 | tetravalent vaccine | N | nd |
| Meizhou1-4 | 35 | tetravalent vaccine | N | nd |
| Meizhou1-5 | 23 | tetravalent vaccine | N | A N T |
| Meizhou1-6 | 52 | tetravalent vaccine | N | A B N |
| Meizhou1-7 | 52 | tetravalent vaccine | N | nd |
| Meizhou1-8 | 21 | tetravalent vaccine | Y | A N T |
| Meizhou1-9 | 24 | tetravalent vaccine | N | A N T P |
| Meizhou1-10 | 50 | tetravalent vaccine | N | nd |
| Meizhou1-11 | 53 | tetravalent vaccine | N | N |
| Meizhou1-12 | 37 | tetravalent vaccine | N | nd |
| Deyang1-1 | 21 | tetravalent vaccine | N | A T P |

to be continued

| **District** | **Age (days)** | **Vaccine** | **Clinic** | **Coccidia species** |
| --- | --- | --- | --- | --- |
| Deyang1-2 | 36 | tetravalent vaccine | N | nd |
| Deyang1-3 | 42 | tetravalent vaccine | N | nd |
| Deyang1-4 | 45 | tetravalent vaccine | N | T |
| Deyang1-5 | 65 | tetravalent vaccine | N | A N T Mi P |
| Deyang1-6 | 45 | tetravalent vaccine | Y | Mi P |
| Deyang1-7 | 59 | tetravalent vaccine | Y | B N Mi P |
| Deyang1-8 | 32 | tetravalent vaccine | N | A B T Mi P |
| Deyang1-9 | 25 | tetravalent vaccine | N | T |
| Deyang1-10 | 56 | tetravalent vaccine | N | T |
| Deyang1-11 | 36 | tetravalent vaccine | Y | Mi |
| Deyang1-12 | 34 | tetravalent vaccine | Y | A N T Mi |
| Deyang2-1 | 27 | tetravalent vaccine | Y | A B T Mi P |
| Deyang2-2 | 44 | tetravalent vaccine | N | B T Mi P |
| Deyang2-3 | 30 | tetravalent vaccine | N | nd |
| Deyang2-4 | 49 | tetravalent vaccine | N | nd |
| Deyang2-5 | 54 | tetravalent vaccine | N | nd |
| Deyang2-6 | 22 | tetravalent vaccine | N | T Ma Mi |
| Deyang2-7 | 24 | tetravalent vaccine | N | nd |
| Deyang2-8 | 38 | tetravalent vaccine | N | nd |
| Deyang2-9 | 24 | tetravalent vaccine | N | Mi |
| Deyang2-10 | 58 | tetravalent vaccine | N | A N |
| Deyang2-11 | 65 | tetravalent vaccine | N | nd |
| Deyang2-12 | 45 | tetravalent vaccine | N | N Mi |
| Deyang3-1 | 26 | trivalent vaccine | N | A T Mi |
| Deyang3-2 | 26 | trivalent vaccine | N | A Mi P |
| Deyang3-3 | 25 | trivalent vaccine | N | A Mi P |
| Deyang3-4 | 49 | trivalent vaccine | Y | Mi P |
| Deyang3-5 | 45 | trivalent vaccine | N | N Mi |
| Deyang3-6 | 45 | trivalent vaccine | N | A B |
| Deyang3-7 | 49 | trivalent vaccine | N | A B Mi P |
| Deyang3-8 | 59 | trivalent vaccine | N | A B Mi |
| Deyang3-9 | 65 | trivalent vaccine | N | nd |
| Deyang3-10 | 59 | trivalent vaccine | N | nd |
| Guiyang1-1 | 49 | tetravalent vaccine | N | A |
| Guiyang1-2 | 34 | tetravalent vaccine | N | A T Mi P |
| Guiyang1-3 | 35 | tetravalent vaccine | N | A N T Mi P |
| Guiyang1-4 | 44 | tetravalent vaccine | N | A B T Mi P |
| Guiyang1-5 | 30 | tetravalent vaccine | N | A Mi |
| Guiyang1-6 | 44 | tetravalent vaccine | N | A T Mi P |
| Guiyang1-7 | 65 | tetravalent vaccine | N | A N |
| Guiyang1-8 | 29 | tetravalent vaccine | N | A N T Mi |

to be continued

| **District** | **Age (days)** | **Vaccine** | **Clinic** | **Coccidia species** |
| --- | --- | --- | --- | --- |
| Guiyang1-9 | 37 | tetravalent vaccine | N | A |
| Guiyang1-10 | 26 | tetravalent vaccine | N | A N T Mi P |
| Guiyang1-11 | 61 | tetravalent vaccine | N | A T |
| Guiyang1-12 | 21 | tetravalent vaccine | N | A N T |
| Guiyang1-13 | 58 | tetravalent vaccine | N | B N |
| Guiyang1-14 | 67 | trivalent vaccine | Y | Ma |
| Guiyang1-15 | 51 | trivalent vaccine | N | A N T Ma Mi |
| Guiyang1-16 | 51 | trivalent vaccine | N | A N Mi P |
| Guiyang1-17 | 47 | trivalent vaccine | N | A N Mi P |
| Guiyang2-1 | 47 | trivalent vaccine | Y | B N Ma Mi P |
| Guiyang2-2 | 25 | trivalent vaccine | N | A T Mi |
| Guiyang2-3 | 25 | trivalent vaccine | N | A T |
| Guiyang2-4 | 26 | trivalent vaccine | N | A T Mi P |
| Guiyang2-5 | 20 | trivalent vaccine | N | A T Mi |
| Guiyang2-6 | 59 | trivalent vaccine | N | A B Mi P |
| Guiyang2-7 | 59 | trivalent vaccine | N | A B Mi P |
| Guiyang2-8 | 59 | trivalent vaccine | N | A T N Mi P |
| Guiyang2-9 | 48 | trivalent vaccine | Y | A T N Ma Mi P |
| Guiyang2-10 | 48 | trivalent vaccine | Y | A B N P |
| Guiyang2-11 | 45 | trivalent vaccine | Y | A N P |
| Kunming1-1 | 28 | tetravalent vaccine | N | nd |
| Kunming1-2 | 44 | tetravalent vaccine | N | A B N T Mi |
| Kunming1-3 | 29 | tetravalent vaccine | Y | A T Mi P |
| Kunming1-4 | 34 | tetravalent vaccine | N | nd |
| Kunming1-5 | 54 | tetravalent vaccine | N | nd |
| Kunming1-6 | 23 | tetravalent vaccine | N | nd |
| Kunming1-7 | 27 | tetravalent vaccine | Y | A |
| Kunming1-8 | 30 | tetravalent vaccine | N | nd |
| Kunming1-9 | 45 | tetravalent vaccine | N | nd |
| Kunming1-10 | 63 | tetravalent vaccine | N | nd |
| Kunming1-11 | 45 | tetravalent vaccine | N | nd |
| Kunming1-12 | 42 | tetravalent vaccine | N | nd |
| Kunming1-13 | 43 | tetravalent vaccine | N | nd |
| Dali1-1 | 61 | tetravalent vaccine | N | nd |
| Dali1-2 | 49 | tetravalent vaccine | N | A B N T Mi |
| Dali1-3 | 45 | tetravalent vaccine | N | A T |
| Dali1-4 | 49 | tetravalent vaccine | N | A P |
| Dali1-5 | 34 | tetravalent vaccine | N | T Mi P |
| Dali1-6 | 63 | tetravalent vaccine | N | A B N T Mi P |
| Dali1-7 | 46 | tetravalent vaccine | N | A T Ma Mi P |
| Dali1-8 | 20 | tetravalent vaccine | N | A N T |

to be continued

| **District** | **Age (days)** | **Vaccine** | **Clinic** | **Coccidia species** |
| --- | --- | --- | --- | --- |
| Dali1-9 | 21 | tetravalent vaccine | N | A N T Mi P |
| Dali1-10 | 57 | tetravalent vaccine | N | A N T P |
| Dali2-1 | 51 | tetravalent vaccine | N | A T |
| Dali2-2 | 32 | tetravalent vaccine | N | A Mi |
| Dali2-3 | 62 | tetravalent vaccine | N | A T Mi |
| Dali2-4 | 26 | tetravalent vaccine | N | A Mi |
| Dali2-5 | 25 | tetravalent vaccine | N | N T P |
| Dali2-6 | 32 | tetravalent vaccine | N | A Mi |
| Dali2-7 | 70 | tetravalent vaccine | N | nd |
| Dali2-8 | 21 | tetravalent vaccine | N | A N T Mi |
| Dali2-9 | 31 | tetravalent vaccine | N | A B N T |
| Dali2-10 | 32 | tetravalent vaccine | N | B |
| Dali2-11 | 56 | tetravalent vaccine | N | A B N T Mi |
