# Supplementary material for: Epidemiological investigation of coccidiosis and associated risk factors in broiler chickens immunized with live anticoccidial vaccines in China
Source: Front Vet Sci. 2024 Mar 19;11:1375026. doi: 10.3389/fvets.2024.1375026 (PMC10986636; doi:10.3389/fvets.2024.1375026)
Supplement: Supplementary file 2 [file Table_2.DOCX]

Supplementary Data 2:

**
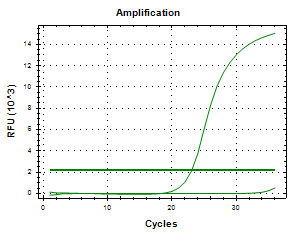
**

Figure S1. Positive and negative results of TB Green real-time PCR test for *E. tenella*.

**
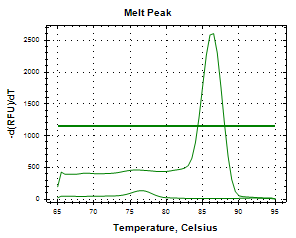
**

Figure S2. The melting peak of positive and negative samples of *E. tenella* real-time PCR test.


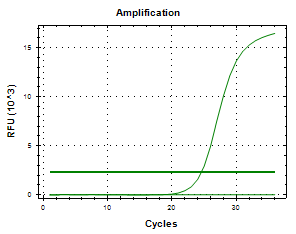


Figure S3. Positive and negative results of TB Green real-time PCR test for *E. necatirx*.


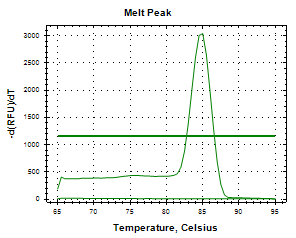


Figure S4. The melting peak of positive and negative samples of *E. necatrix* real-time PCR test.


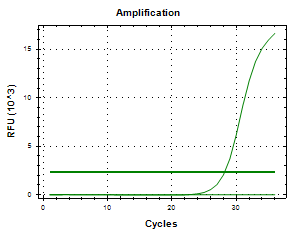


Figure S5. Positive and negative results of TB Green real-time PCR test for *E. maxima*.


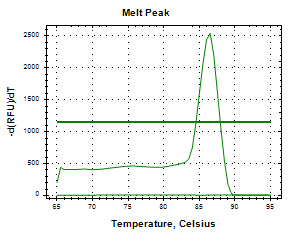


Figure S6. The melting peak of positive and negative samples of *E. maxima* real-time PCR test.


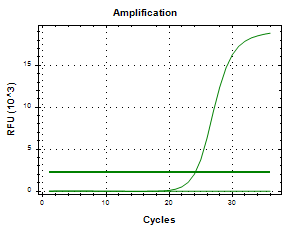


Figure S7. Positive and negative results of TB Green real-time PCR test for *E. acervulina*.


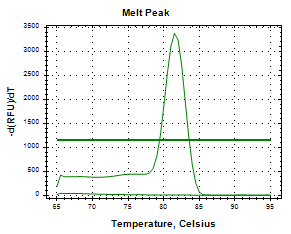


Figure S8. The melting peak of positive and negative samples of *E. acervulina* real-time PCR test.


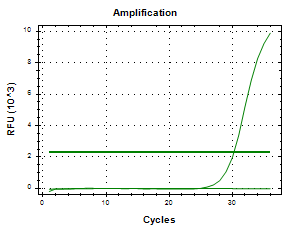


Figure S9. Positive and negative results of TB Green real-time PCR test for *E. brunetti*.


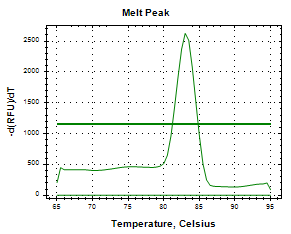


Figure S10. The melting peak of positive and negative samples of *E. brunetti* real-time PCR test.


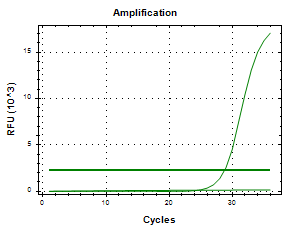


Figure S11. Positive and negative results of TB Green real-time PCR test for *E. mitis*.


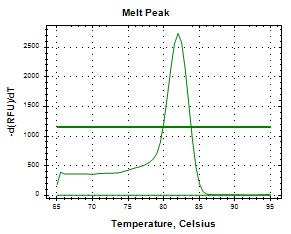


Figure S12. The melting peak of positive and negative samples of *E. mitis* real-time PCR test.

**
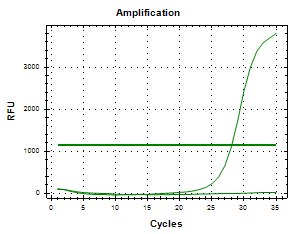
**

Figure S13. Positive and negative results of TB Green real-time PCR test for *E. praecox*.

**
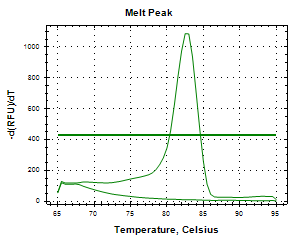
**

Figure S14. The melting peak of positive and negative samples of *E. praecox* real-time PCR test.
